# High-κ Wide-Gap Layered Dielectric for Two-Dimensional van der Waals Heterostructures

**DOI:** 10.1021/acsnano.3c10411

**Published:** 2024-04-01

**Authors:** Aljoscha Söll, Edoardo Lopriore, Asmund Ottesen, Jan Luxa, Gabriele Pasquale, Jiri Sturala, František Hájek, Vítězslav Jarý, David Sedmidubský, Kseniia Mosina, Igor Sokolović, Saeed Rasouli, Tibor Grasser, Ulrike Diebold, Andras Kis, Zdeněk Sofer

**Affiliations:** † Department of Inorganic Chemistry, University of Chemistry and Technology Prague, Technicka 5, 166 28, Prague 6, Czech Republic; ‡ Institute of Electrical and Microengineering, École Polytechnique Fédérale de Lausanne (EPFL), CH-1015 Lausanne, Switzerland; § Institute of Materials Science and Engineering, École Polytechnique Fédérale de Lausanne (EPFL), CH-1015 Lausanne, Switzerland; ∥ Institute of Physics of the Czech Academy of Sciences, v.v.i., Cukrovarnická 10, 162 00, Prague 6, Czech Republic; ⊥ Institute of Microelectronics, TU Wien, Gußhausstraße 27−29, 1040 Vienna, Austria; ○ Institute of Applied Physics, TU Wien, Wiedner Hauptstraße 8−10, 1040 Vienna, Austria

**Keywords:** dielectric, high-k, two-dimensional materials, crystal synthesis, field-effect transistors, heterostructures, excitons

## Abstract

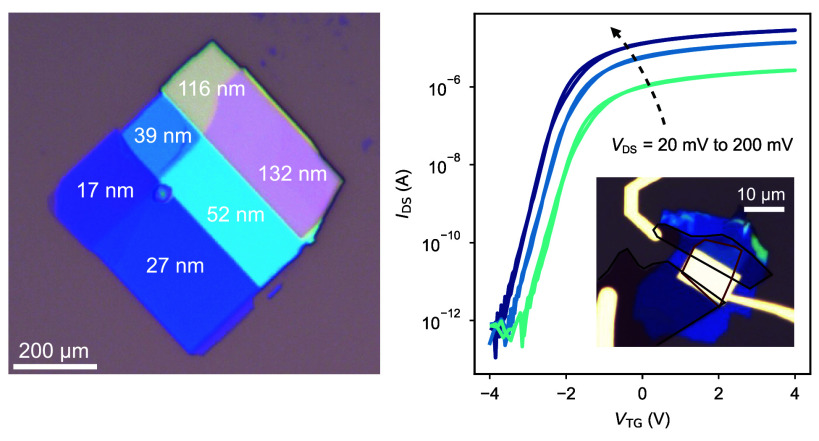

van der Waals heterostructures of two-dimensional materials have unveiled frontiers in condensed matter physics, unlocking unexplored possibilities in electronic and photonic device applications. However, the investigation of wide-gap, high-κ layered dielectrics for devices based on van der Waals structures has been relatively limited. In this work, we demonstrate an easily reproducible synthesis method for the rare-earth oxyhalide LaOBr, and we exfoliate it as a 2D layered material with a measured static dielectric constant of 9 and a wide bandgap of 5.3 eV. Furthermore, our research demonstrates that LaOBr can be used as a high-κ dielectric in van der Waals field-effect transistors with high performance and low interface defect concentrations. Additionally, it proves to be an attractive choice for electrical gating in excitonic devices based on 2D materials. Our work demonstrates the versatile realization and functionality of 2D systems with wide-gap and high-κ van der Waals dielectric environments.

The International Roadmap for Devices and Systems (IRDS) has identified two-dimensional semiconductors as the channel materials for future technology nodes in the coming decade.^1^ 2D materials have been proven to be compatible with standard semiconductor fabrication methods including the CMOS process.^2^ Moreover, they have revealed numerous possibilities in noncomputational systems under the more-than-Moore paradigm, ranging from back-end-of-the-line (BEOL) to cointegration with silicon technology in monolithic solutions.^3^

The scaling of silicon technology has led to the transition of insulating materials for electrical gating in transistor devices from SiO_2_ to high-κ dielectrics. However, the integration of 2D materials with 3D high-κ dielectrics, such as Al_2_O_3_ and HfO_2_, is challenging due to the presence of dangling bonds at their interface, which adversely impact device performance.^4^ Even though high temperature treatment or the use of seeding layers have resulted in the improved performance of electrical devices based on atomic-layer-deposited (ALD) oxides,^5^ the achievement of ideal interfaces remains a work in progress.

Native high-κ oxides, obtained by oxidizing a suitable 2D semiconductor, are expected to offer ideal dielectric interfaces,^6^ but their application is limited to semiconductors that allow for native oxide growth. High-κ perovskite films have also been transferred onto 2D materials, demonstrating gate dielectric functionality with van der Waals interfaces.^7,8^ However, these alternatives lack the modularity enabled by fully van der Waals heterostructures. In fact, a key strength of using 2D materials for device fabrication is the possibility of stacking them into heterostructures without critical structural constraints.^9^ This is important for exploring electrical and optical properties, as well as emergent physical effects, thanks to the combination of different 2D materials in freely configurable stacks.

Hexagonal boron nitride (hBN) has played a crucial role in these advancements as the primary layered dielectric to be successfully employed in van der Waals heterostructures of 2D materials. However, the static dielectric constant of hBN is limited to a mediocre permittivity of about 5.^4^ Therefore, high-κ-layered dielectrics are required as an essential step toward the future scaling of a fully-2D MOSFET platform. Furthermore, layered dielectrics are used in optics and optoelectronics as encapsulation layers for optically active 2D materials, such as TMDCs, since the dielectric environment is known to heavily influence their excitonic properties.^10^ However, until now, hBN has been the only 2D dielectric that has been successfully employed as an encapsulant in excitonic devices based on van der Waals heterostructures. Therefore, the synthesis of other 2D dielectrics could provide grounds for exploration to modulate the effects of the dielectric environment in excitonic devices based on 2D materials.

Recently, Bi_2_SeO_5_ has been synthesized as a single crystal high-κ layered material for use as an insulator.^11^ However, its low bandgap of *E*_g_ ≃ 3.6 eV critically limits its possible applications as a versatile dielectric in 2D field-effect structures, since its *E*_g_ is comparable to that of semiconducting materials such as InSe, GaSe, and GaS.^12^ In fact, in the search for high-κ dielectrics, versatile gate insulators require a band alignment with barriers of 1 eV for both electrons and holes.^13^ Considering that most TMDC bandgaps are around 2 eV and that metal monochalcogenides exhibit gaps up to 3 eV,^12^ a van der Waals high-κ dielectric with *E*_g_ > 5 eV is highly desirable to enable wide material-independent van der Waals integration in 2D heterostructures. Rare-earth oxyhalides have been theoretically predicted to be easily exfoliable layered insulators with a wide bandgap and high-κ both in bulk and monolayer forms.^14^ Moreover, they have been indicated as good candidates as insulating media for TMDC devices thanks to the negligible interfacial charge transfer and sizable band offsets with such materials.^15^ In this work, we synthesize crystals of LaOBr, a rare-earth oxybromide that has been indicated as an ideal layered dielectric for 2D heterostructure devices offering low leakage currents and a wide bandgap.^14^ Here, we develop a high-temperature flux growth method for LaOBr that focuses on the production of crystals in the form of platelets, and we provide its complete bulk characterization, highlighting its bulk bandgap of approximately 5.3 eV.

We reveal the layered structure of LaOBr that can be readily cleaved to expose a Br-terminated (001) surface. We further exfoliate LaOBr and employ the established pick-and-place fabrication techniques to build van der Waals heterostructures with LaOBr as the dielectric material. By conducting electrical transport measurements in graphene field-effect structures, we determine the static out-of-plane dielectric constant to be approximately equal to 9. Subsequently, we employ LaOBr as a gate dielectric for transistor operation in a field-effect structure with MoS_2_. We observe a low subthreshold slope (∼85 mV dec^–1^) and a corresponding reasonably low interface defect concentration (*D*_it_ ≃ 1.06 × 10^12^ cm^–2^ eV^–1^), together with a high on–off ratio (*I*_on_/*I*_off_ > 10^8^), low leakage current (<10^–4^ A cm^–2^ for bulk flakes up to 1.5 MV cm^–1^), and small *I*–*V* hysteresis. These results provide the evidence that LaOBr can be used as a high-κ dielectric in high-performance, fully van der Waals electronic devices. Furthermore, we demonstrate that LaOBr can be employed as encapsulation and gate insulator for excitonic devices based on 2D materials, showing the modulation of excitonic species in MoSe_2_ by electronic gating.

This study highlights the promising potential of LaOBr in advancing the field of 2D heterostructures and their applications in electronic and optoelectronic devices.

## Results and Discussion

### LaOBr Crystal Synthesis

Single-crystal growth of rare-earth oxyhalides has been scarcely documented in the literature until now. In particular, the synthesis of LaOBr in crystal form has been previously achieved using anhydrous LaBr_3_ and La_2_O_3_ with an excess of LaBr_3_ as a flux, leading primarily to the formation of LaOBr as a mixture of needles and narrow platelets.^16^ Although we were able to successfully replicate this growth (Supporting Information, Note 1), the predominance of needles is problematic for van der Waals heterostructure fabrication. Notably, needles of LaOBr tend to grow perpendicularly to the c-plane, likely along a screw dislocation, resulting in a minimal (001) cross-section, making them unsuitable for exfoliation (Supporting Information, Figure S1). Furthermore, the previously developed synthesis is performed in a sealed quartz ampule, which significantly reduces its accessibility and introduces potential hazards stemming from gas production during the synthesis.

**Figure 1:**
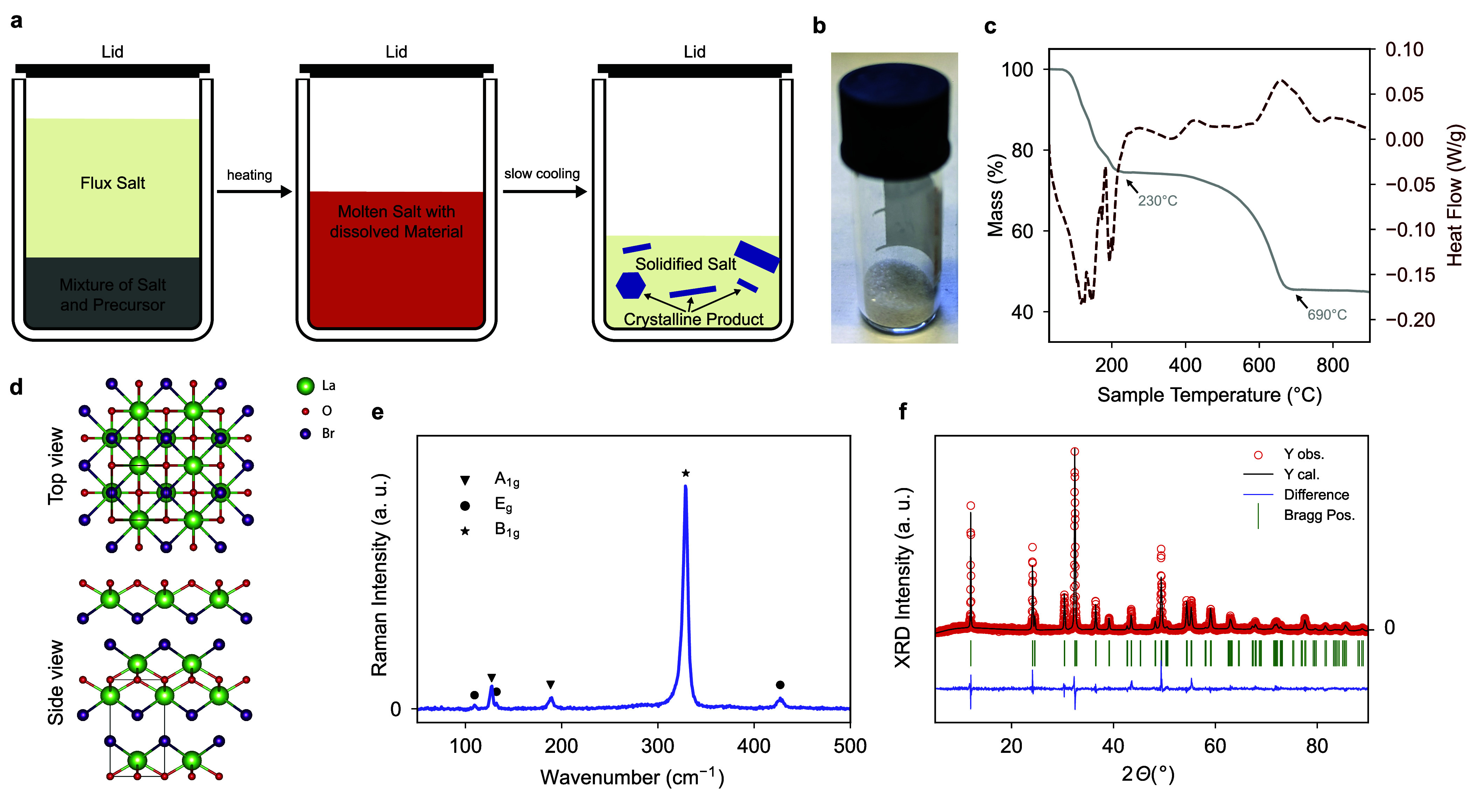
Synthesis and characterization of bulk LaOBr. (a) Synthesis schematic for the growth of LaOBr single crystals for the method used in this work. (b) LaOBr bulk crystals obtained from the synthesis. (c) TGA-DSC measurements of LaBr_3_·7H_2_O in Ar/O_2_ atmosphere shows gradual conversion into anhydrous LaBr_3_ completed at 230 °C, and finally LaOBr completed at 690 °C. (d) Refined structure of LaOBr as obtained from XRD, visualized with VESTA^45^ (e) Raman spectroscopy on bulk LaOBr. We assigned two A_1g_ modes, three E_g_ modes, and one B_1g_ mode based on DFT calculations (Supporting Information, Note 4) and in accordance with the literature:^18,19^ (f) X-ray powder diffractogram (XRD) of bulk LaOBr with experimental data in red, Rietveld refinement fit in black, literature peak positions in green, and difference in blue.^16^

In order to devise a more suitable synthesis of LaOBr (Figure 1a,b), we conducted thermogravimetric analysis (TGA) coupled with differential scanning calorimetry (DSC) on LaBr_3_·7H_2_O in a dynamic argon atmosphere. The resulting TGA is shown in Figure 1c, and a more detailed description is given in Supporting Information, Note 2. The TGA-DSC analysis indicates that, contrary to the procedure established by Haeuseler et al.,^13^ we do not require the use of anhydrous LaBr_3_ in our method, as the crystal water is eliminated during the reaction when we use an open vessel. Moreover, this procedure eliminates the need for adding La_2_O_3_ since atmospheric oxygen is sufficient as the main source of oxygen (Supporting Information, Figure S2).

Therefore, our modified LaOBr growth method takes place in an open corundum crucible under atmospheric conditions using a eutectic mixture of alkaline and earth-alkaline metal-salts. We chose a mixture of NaBr and MgBr_2_ as a flux with a low melting point to maximize the temperature range for material growth. By avoiding quartz ampules, we further aim to increase the accessibility of LaOBr thanks to its improved growth reproducibility (Methods and Supporting Information, Note 3).

**Figure 2:**
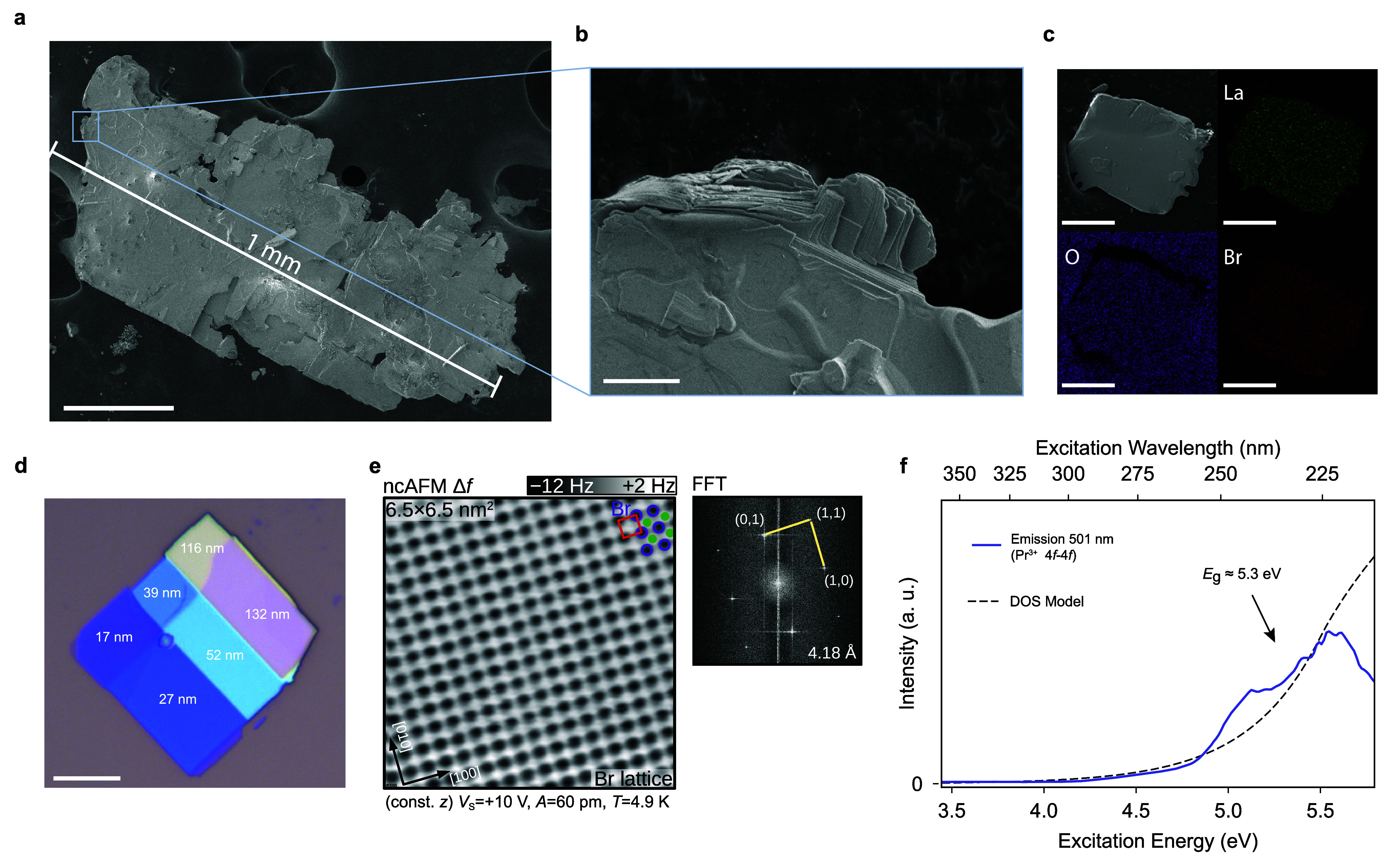
Characterization of LaOBr crystals and exfoliated flakes. (a) SEM image of a LaOBr single crystal with a lateral dimension of 1 mm obtained from the synthesis method used in this work. Scale bar: 200 μm. (b) The magnified view of the crystal edge shows the terraced morphology indicative of a layered structure. Scale bar: 10 μm. (c) EDS map on a single crystal of LaOBr, showing uniform distribution of elements across the whole crystal. Scale bar: 50 μm. (d) Flake of LaOBr on a SiO_2_ substrate (270 nm), obtained following the standard tape exfoliation commonly employed for 2D materials and van der Waals heterostructure fabrication. Large flakes of tens of micrometers in width are easily obtained. The different colors of the flake terraces on our substrate are related to different heights, with increasing values from dark blue to light blue (10–60 nm), toward yellow and pink (>100 nm). The height measurements were obtained by tapping mode AFM, as shown in Figure S5 in the Supporting Information. Scale bar: 10 μm. (e) (Left) Atomically resolved, constant-height ncAFM image of a LaOBr crystal cleaved and studied in UHV. A lattice of Br atoms imaged in attraction (frequency shift Δ*f* further from zero), and sketched in purple, terminates a cleaved LaOBr (001) surface, while the near-subsurface La lattice, sketched in green, is not visible (Δ*f* closer to zero). (Right) The FFT of an atomically resolved image measures a cubic unit cell of 4.18 Å, also indicated in red in the ncAFM image. (f) Photoluminescence excitation measurement on LaOBr crystals compared to a density of states (DOS) model, as further detailed in Supporting Information, Note 6. The emission wavelength is set to 501 nm (2.47 eV), corresponding to the 4*f*–4*f* transition of praseodymium impurity. We calculate the bandgap as 5.3 eV (Supporting Information, Note 6).

After performing the synthesis at 1000 °C and separating the material from the flux, we obtained LaOBr in the form of colorless platelets with lateral dimensions of up to 1 mm (Figure 2a). Utilizing this method, we successfully eliminated the formation of needles, yielding only thin platelets of LaOBr that are highly suitable for exfoliation (Supporting Information, Figure 3).

### LaOBr Bulk Characterization

We verified the phase purity of the obtained LaOBr platelets by using powder X-ray diffraction (XRD), as shown in Figure 1f. LaOBr crystallizes in the tetragonal space group *P*4/*nmm* with no additional phases. Its lattice constants were calculated from Rietveld refinement as *a* = *b* = 4.1618 Å, and *c* = 7.3813 Å (α = β = γ = 90°). Figure 1d shows the crystal structure obtained from the refinement, revealing a clear van der Waals gap perpendicular to the *c*-axis. We note that LaOBr exhibits a tetragonal symmetry, an uncommon property among van der Waals materials, most of which possess a hexagonal symmetry.^9,17^ Furthermore, a monolayer of the material comprises 5 rows of atoms, in contrast to TMDCs with 3 rows of atoms, or hBN and graphene which are a single atom thick.

As shown in Figure 1e, we performed Raman spectroscopy on the bulk sample. We assigned the two A_1g_ modes to 127.3 cm^–1^ (A_1g1_) and 188.5 cm^–1^ (A_1g2_). We assigned the three E_g_ modes to 109.5 cm^–1^ (E_g1_), 132.3 cm^–1^ (E_g2_), and 427.1 cm^–1^ (E_g3_), and the intense (B_1g_) mode to 329.0 cm^–1^ (B_1g1_), based on DFT calculations (Supporting Information, Note 4) and in accordance with the literature.^18,19^ Furthermore, we analyzed the surface composition by X-ray photoelectron spectroscopy (XPS), demonstrating the absence of surface impurities. The corresponding XPS spectra and obtained binding energies are discussed in detail in Supporting Information, Note 5.

We characterized the sample morphology using scanning electron microscopy (SEM), equipped with an energy dispersive X-ray spectrometer (EDS). Based on SEM measurements, we observed that LaOBr forms thin platelets with lateral dimensions ranging from 100 μm to 1 mm (Figure 2a) and approximate thicknesses of around 5–20 μm. Many of the platelets display a terraced morphology on their edges, which is typical for layered structures (Figure 2b, Supporting Information, Figure S3). The EDS analysis confirms the correct stoichiometry of the samples, with an atomic ratio of 33 ± 1% for each element. Additionally, the EDS map taken from a single crystal of LaOBr reveals a uniform distribution of all three elements across the entire crystal (Figure 2c).

LaOBr single crystals were successfully cleaved inside an ultrahigh vacuum (UHV) chamber following a standard procedure for layered materials (Methods). Cleaved LaOBr (001) surfaces exhibit atomically flat surfaces, as revealed by noncontact atomic force microscopy (ncAFM) in Figure 2e. The observation of well-ordered lattices indicates that cleavage (exfoliation by extension) creates two mirror-symmetric Br-terminated surfaces resulting from cleavage along the van der Waals gap (Figure 1d). Cleaving any other (001) plane would result in surfaces with 0.5 ML of less-ordered or disordered under-coordinated ions.^20^ A cubic unit cell of 4.18 ± 0.03 Å was measured by the two-dimensional fast Fourier transform (FFT) of an atomically resolved ncAFM image, with a 1.3° asymmetry due to the experimental distortions. No tunneling current was observed with a 10 fA precision after applying a 10 V potential difference in a very close proximity (<5 Å) of the tip to the sample (Figure 2e).

To determine the bandgap of bulk LaOBr, we performed photoluminescence excitation spectroscopy (PLE), which yielded a value of 5.3 eV (Figure 2f), although a large value of the Urbach tail energy was obtained (0.39 eV). Further details can be found in Supporting Information, Note 6. This value is in agreemend with previous measurements obtained using UV–vis spectroscopy.^21^

### Dielectric Constant Estimation

Like other rare-earth oxyhalides, LaOBr exhibits a significant difference between its optical and static dielectric constants.^14^ Here, we focus on the optical (ϵ_∞_) and static (ϵ_0_) dielectric constants in the out-of-plane direction (⊥), which are particularly relevant for identifying and using LaOBr as a layered gate dielectric. The out-of-plane optical dielectric constant, which considers only the electronic response, was calculated to be ϵ_∞,⊥_ ∼ 4.6 for LaOBr.^14^ This value is important for identifying LaOBr flakes on a substrate since the optical refractive index of the material is given by its optical dielectric constant. Due to its bandgap of *E*_g_ ∼ 5.3 eV and low optical dielectric constant of 4.6, LaOBr can be easily distinguished on SiO_2_ and PDMS substrates, commonly used for the exfoliation of 2D materials. Moreover, since the optical dielectric constant of LaOBr is close to that of hBN,^14^ its thickness can be estimated by color contrast in a similar manner as it is done for hBN.^22^Figure 2d shows the identification of various thicknesses of LaOBr on SiO_2,_ based on AFM measurements (Supporting Information, Figure S6). LaOBr flakes tend to exhibit rectangular shapes due to their tetragonal crystal symmetry, as shown in Figure 2d and in Supporting Information, Figure S6. We note that the ease of exfoliation and identification of the 2D dielectric material, along with the ability to estimate its thickness by optical means, are crucial factors for successfully incorporating the high-κ dielectric in the fabrication of van der Waals heterostructures based on the most established substrates and techniques.

**Figure 3:**
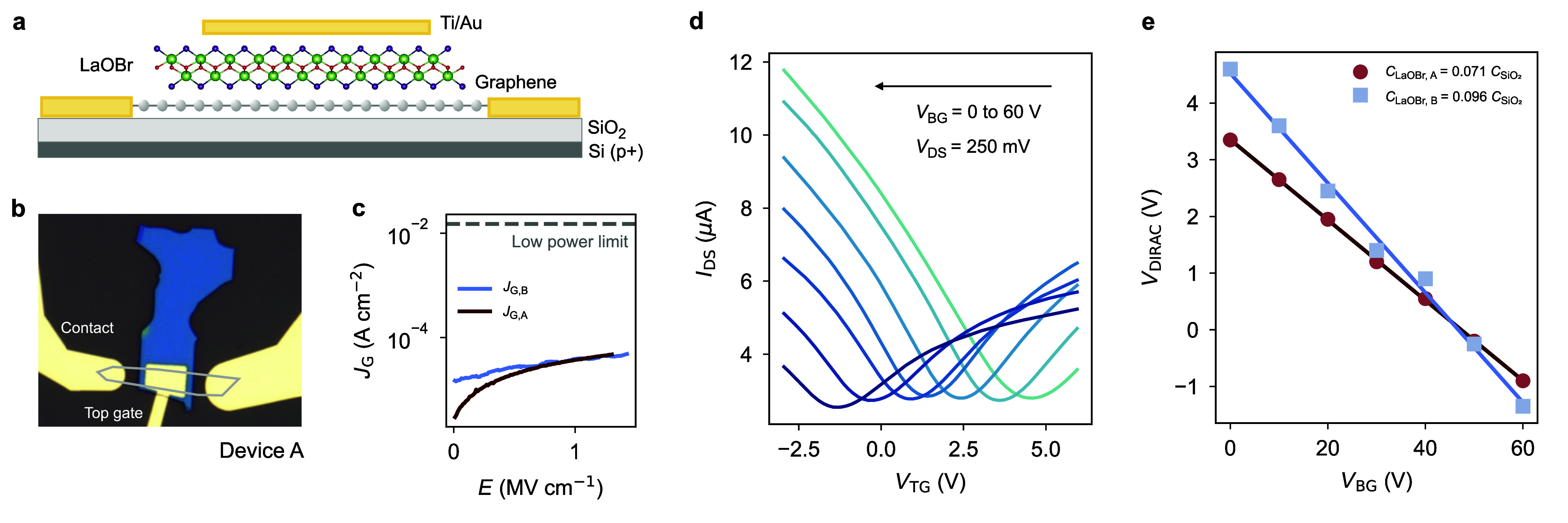
LaOBr dielectric constant and leakage current. (a) Schematic of the graphene field-effect devices used for the estimation of the dielectric constant of LaOBr. Graphene is contacted by lateral Ti/Au electrodes, with a top LaOBr dielectric and a Ti/Au top gate. The bottom dielectric is SiO_2_ (270 nm) over highly p-doped Si, used as bottom gate. (b) Optical micrograph of device A, showing the bulk LaOBr flake in blue and the graphene layer highlighted in gray. The Ti/Au lateral electrodes and top gate are labeled. (c) Gate leakage current as a function of vertical electric field in the field-effect graphene devices A and B, showing leakage currents (<10^–4^ A cm^–2^) lying orders of magnitude below the low-power limit (1.5 × 10^–2^ A cm^–2^). The back gate voltage is kept at *V*_BG_ = 30 V as a reference, while comparable results are obtained independently on *V*_BG_. (d) Drain-source current in device A as a function of the applied top gate voltage *V*_TG_ for bottom gate voltages in the range 0 < *V*_BG_ < 60 V. The drain-source bias is fixed at *V*_DS_ = 250 mV. The minima of the curves are obtained when the Fermi level lies at the graphene Dirac point position *V*_DIRAC_. (e) Change of *V*_DIRAC_ with respect to *V*_BG_ for devices A and B. The different slopes of the curves are related to the different thicknesses of the LaOBr layers, with 56 and 46 nm for devices A and B, respectively. The obtained capacitance ratios are highlighted in the legend of the plot. From these ratios, we use eq 1 to extract out-of-plane static dielectric constants of 8.6 and 9.4 for devices A and B, respectively, giving an average estimate of ϵ_0,⊥_ ≃ 9. All measurements in this figure were taken at room temperature in high vacuum (10^–6^ mbar).

On the other hand, the static dielectric constant ϵ_0,⊥_, comprising both ionic and electronic responses, is the key reference value for electrical gating in field-effect structures. Specifically, the out-of-plane static dielectric constant of LaOBr has been theoretically predicted to be 12.5 in its bulk form, with a slightly higher value for the monolayer case.^14^ To determine ϵ_0,⊥_ by electrical measurements, we utilize graphene field-effect dual-gated devices (Figure 3a,b). We fabricate fully van der Waals field-effect heterostructures using an established dry pick-up technique, showcasing the exfoliable and stackable nature of LaOBr as a 2D dielectric (Methods, Supporting Information, Figure S7).

The Dirac point of graphene in field-effect structures corresponds to the position of maximum lateral resistance in gate sweeps with a fixed bias voltage (Figure 3d). By performing dual-gate voltage sweeps, we can compare the capacitive field-effect modulation from the bottom and top gate dielectrics, represented by SiO_2_ and LaOBr, respectively.^8,23^ We use SiO_2_ as an established reference insulator with ϵ_0,⊥_^SiO2^ ∼ 3.9. Therefore, the slope of the change in the Dirac point in Figure 3e is directly determined by the ratio of the bottom and top gate capacitances:
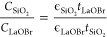
1Our bottom substrate oxide has a fixed thickness of *t*_SiO2_ = 270 nm, while the LaOBr flakes have a measured thickness of *t*_LaOBr,A_ = 46 nm and *t*_LaOBr,B_ = 56 nm, as dermined by AFM. The different slopes observed in the linear fit of the Dirac point shift are due to the varying thickness of LaOBr. From eq 1, we calculate the average out-of-plane static dielectric constant of LaOBr to be ϵ_0,⊥_ ≃ 9 ± 0.4 (Supporting Information, Figure S8). This value aligns within 25% of the theoretically calculated value of 12.5, and the uncertainty range falls within the common margin of calculations for other low-κ van der Waals dielectrics.^14^ With a measured bandgap of 5.3 eV and a static dielectric constant of 9, LaOBr is a 2D equivalent of commonly employed 3D oxides such as Al_2_O_3_ (Supporting Information, Figure S9), with the crucial feature of full integration with van der Waals systems.

Additionally, we measured the gate leakage current in our graphene field-effect devices with LaOBr, finding values that are orders of magnitude lower than both the gate limit and the low-power limit^13^ for a range of electric fields up to 1.5 MV cm^–1^ (Figure 3c). Furthermore, we assessed the breakdown of our LaOBr flakes using metal–insulator–metal (MIM) structures, obtaining an average breakdown field value of approximately *E*_BD_ ≃ 8 MV cm^–1^ for different bulk thicknesses by Poole-Frenkel plots (Supporting Information, Figure S10). We note that such an extraction is an approximate estimate of the breakdown field of bulk LaOBr flakes. The measured breakdown is comparable to that of hBN^24^ and of other commonly used 3D bulk oxides as Al_2_O_3_.^25^ These results strongly suggest that LaOBr can be used as a high-κ and low-leakage layered dielectric material, which aligns well with theoretical predictions.^14^

### Fully van der Waals High-κ Field-Effect Transistor

**Figure 4:**
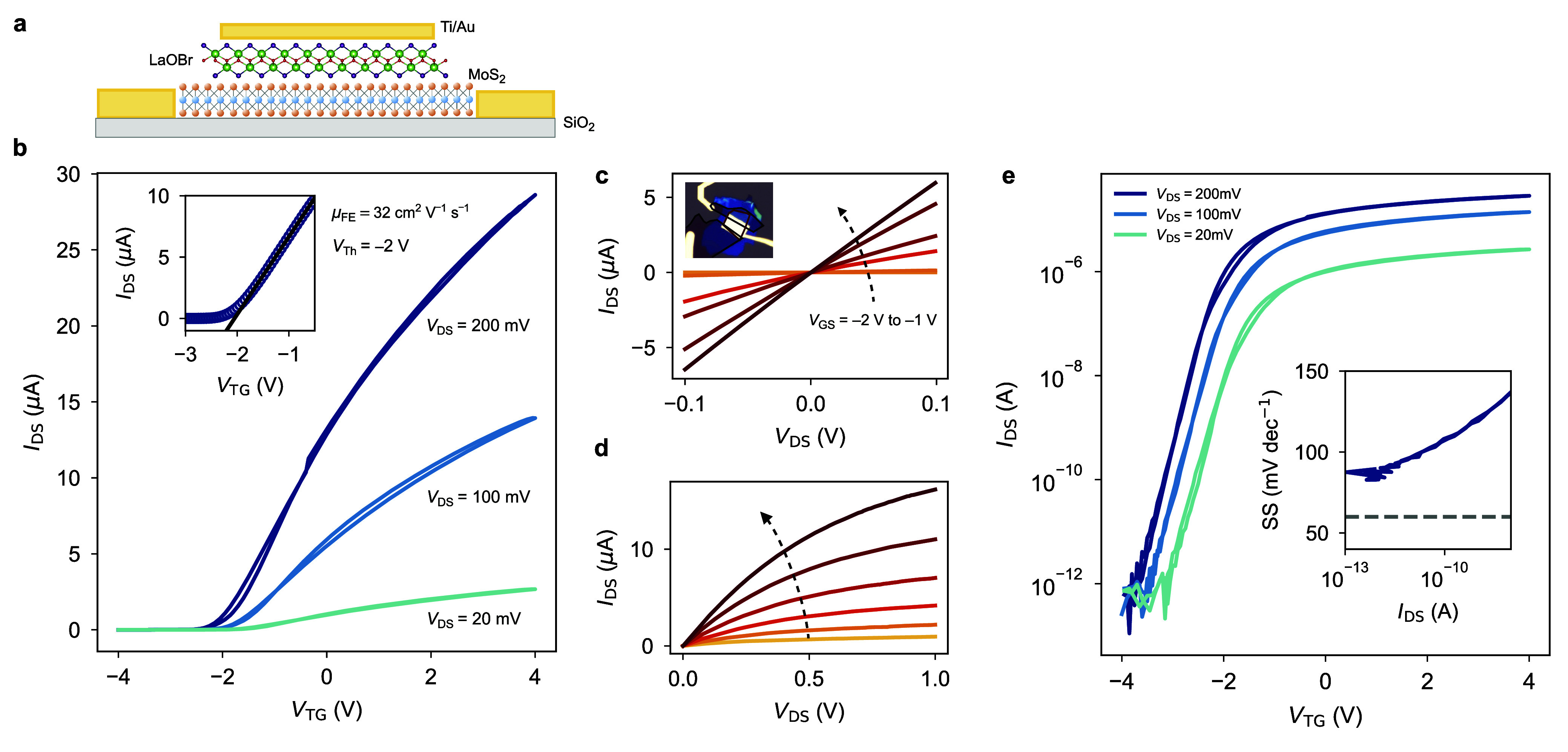
MoS_2_ field-effect transistor with LaOBr. (a) Schematic of the MoS_2_ field-effect transistor with LaOBr as gate dielectric, with source-drain lateral Ti/Au electrodes and a top Ti/Au gate. (b) Drain-source current *I*_DS_ of the 4-layer MoS_2_ transistor with LaOBr as the gate dielectric as a function of the applied top gate voltage *V*_TG_. All top-gate scans were taken with a speed lower than 25 mV/s. The channel width and length of the transistor are *W* ≃ 8.8 μm and *L* ≃ 3.6 μm, respectively, with a dielectric thickness of *t*_LaOBr_ ≃ 20 nm. Three different bias conditions *V*_DS_ are reported and labeled on the curves. Inset: linear region of the transfer characteristic for *V*_DS_ = 200 mV, with an extracted threshold voltage *V*_Th_ ≃ −2 V and two-terminal field-effect mobility μ_FE_ ≃ 32 cm^2^ V^–1^ s^–1^. (c) *I*_DS_ as a function of *V*_DS_ for gate voltages in the range −2 V < *V*_GS_ < −1 V. The range of bias is kept as −0.1 V < *V*_DS_ < 0.1 V to highlight the linearity of the curves at small bias, an indication of ohmic contacts. Inset: optical micrograph of the transistor device, with the LaOBr flake (20 nm) in blue, while the few-layer graphite drain/source flakes and the MoS_2_ flake are highlighted in black and red, respectively. The connections to the few-layer graphite flakes, as well as the top gate, are all made with Ti/Au. (d) Output characteristics of the transistor for −2 V < *V*_GS_ < −1 V, showing a clear modulation from linear to saturation regions. (e) Transfer characteristics from (a) plotted in logarithmic scale, highlighting the subthreshold and above-threshold regions for all biases. Inset: subthreshold slope from the local derivative ∂*V*_TG_/∂*I*_DS_ as a function of *I*_DS_, with minimum values around 85 mV dec^–1^. All measurements in this figure were taken at room temperature in high vacuum (10^–6^ mbar).

To showcase the applicaiton of LaOBr as a high-κ insulator for fully van der Waals devices, we fabricated a field-effect transistor using few-layer MoS_2_ as the channel material and a 20 nm thick LaOBr flake as the gate dielectric (Figure 4a, Supporting Information, Figure S11). Figure 4b shows the room-temperature transfer characteristics obtained by sweeping the top-gate voltage *V*_TG_ with a fixed bias *V*_DS_. All sweeps are performed in both directions of the *V*_TG_ scale. In the inset of Figure 4b, we focus on the forward sweep with *V*_DS_ = 200 mV, revealing a threshold voltage of *V*_Th_ ≃ −2 V and a field-effect two-terminal mobility of μ_FE_ ≃ 32 cm^2^ V^–1^ s^–1^. The obtained mobility is comparable with the previously reported two-terminal μ_FE_ obtained for few-layer MoS_2_ with lateral few-layer graphite contacts.^8^

Hysteretic behaviors in transfer curves are commonly observed in 2D material field-effect transistors based on bulk 3D oxides.^26^ These nonidealities are generally ascribed to oxide traps. However, in our case, all transfer curves in Figure 4b exhibit a small hysteresis on the order of tens of mV, indicating a low defect density at the interface between LaOBr and MoS_2_. Moreover, we measured the output characteristics of the LaOBr/MoS_2_ transistor by sweeping the bias voltage *V*_DS_ with a fixed value of *V*_TG_ (Figure 4c,d). Figure 4c shows a highly linear behavior at low bias voltages independent of the gate modulation, indicating the absence of Schottky barrier effects. The field-effect modulation of the channel conductance reveals the conventional linear and saturation regimes of 2D MOSFET devices (Figure 4d). Additionally, in Figure 4e, we show the transfer curves in logarithmic scale to highlight the subthreshold trend, and we observe a subthreshold slope as low as 85 mV/dec This value is directly related to the interface defect density *D*_it_ by , and we estimate it to be *D*_it_ ≃ 1.06 × 10^12^ cm^–2^ eV^–1^, which is comparable to other high-performing devices based on MoS_2_.^4^ Therefore, the small hysteresis and the low subthreshold slope indicate an ideal van der Waals interface between LaOBr and MoS_2_. In particular, the estimated defect density is higher than the best results obtained for exfoliated MoS_2_ flakes on hBN^27^ and passivated SiO_2_^28^ (<10^11^ cm^–2^ eV^–1^), while being well comparable with MoS_2_ on other high-k dielectrics, as Al_2_O_3_^29^ and Ta_2_O_5_^30^ (∼10^12^ cm^–2^ eV^–1^), as well as other 2D dielectrics. A thorough comparison between LaOBr and other known layered dielectrics can be found in Supporting Information, Note 7 for completeness.

### Exciton Control with LaOBr

**Figure 5:**
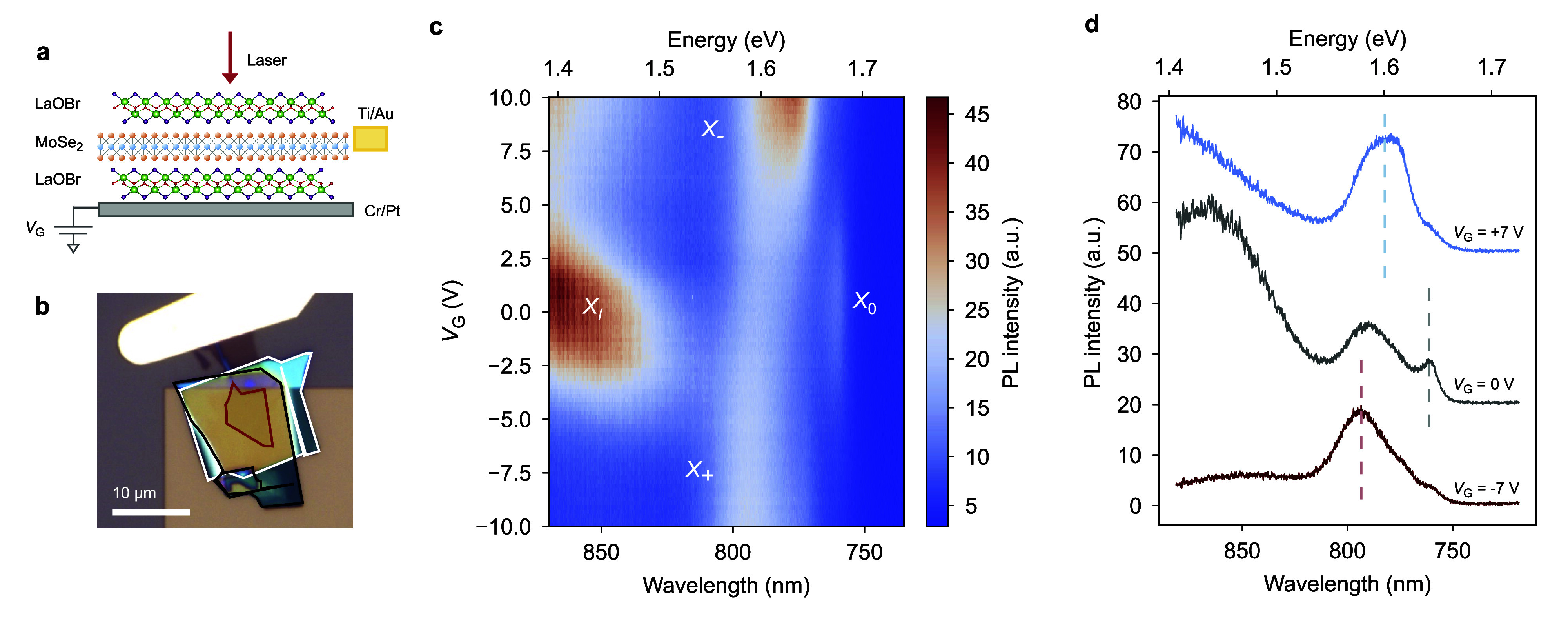
Gate modulation of excitonic species in LaOBr-encapsulated monolayer MoSe_2_. (a) Schematic of the heterostructure based on monolayer MoSe_2_ encapsulated with LaOBr on a Cr/Pt bottom gate. The semiconductor is contacted by a Ti/Au electrode. The structure is excited by a red laser (640 nm) focused at the diffraction limit with a power of 100 μW. (b) Optical micrograph of the excitonic device, showing the monolayer MoSe_2_ flake (red), together with the top (white) and bottom (black) LaOBr flakes. The Cr/Pt gate and the Ti/Au contact to MoSe_2_ are clearly visible. Scale bar: 10 μm. (c) Gate-dependent PL emission of the excitonic species in LaOBr-encapsulated monolayer MoSe_2_. Neutral, charged (trions), and impurity-bound species are evidenced. (d) Line cuts of PL emission at negative (red), zero (gray), and positive (blue) gate voltages. Neutral, negative, and positive trion PL emission peaks are centered at approximately 1.63, 1.60, and 1.57 eV in energy. The broad impurity-bound exciton observed at lower energies is significantly quenched with increasing electrostatic doping, with predominant trion formation.

The electrical control of excitonic features in optically active 2D materials has significant implications for both fundamental discoveries and applications in the field of optoelectronics.^31,32^ In particular, van der Waals dielectric encapsulation provides protection and enables electrical gating of optically-active 2D materials, such as TMDCs, facilitating the observation of charged excitonic species known as trions. Here, we fabricated a heterostructure with monolayer MoSe_2_ fully encapsulated by LaOBr on a bottom metal gate (Figure 5a,b). In Figure 5c,d, we present the gate-dependent excitonic features in our device, measured by photoluminescence (PL) spectroscopy at a temperature of 4 K (Methods). The presence of an impurity-bound state *X*_I_ is aligned with what is generally obtained for high-quality TMDCs on different substrates.^33,34^

From the measured spectra, we observe that the *X*_0_ line shape is narrower with respect to the *X*_+_ and *X*_–_ trions. We note that the trion peaks feature lineshapes as broad as those usually obtained on bare SiO_2_, in the order of tens of meV.^33,35^ In particular, the non-Lorentzian shape of the trion PL features, together with the appearance of a sizable signal even at charge neutrality, suggest the presence of a resonance between the excitonic transitions and the quasi-continuum of states following the Fano effect.^36^ We can attribute these observations to the presence of electrostatic disorder between the LaOBr and the MoSe_2_ flakes, which is further indicated by the fact that the defective *X*_*I*_ PL emission intensity is stronger than that of *X*_0_ at charge neutrality.^33^ It is also known that inhomogeneous broadening of excitons in TMDCs can be caused by local variabilities in the dielectric environment.^10^ Thus, the presence of inhomogeneous broadening in our features could indicate that the dielectric is not perfectly conformal to the TMDC, consistent with the complex molecular structure of LaOBr (Figure 1d).

We note that further theoretical and experimental studies are needed to understand electrostatic disorder and the manipulation of excitonic states with LaOBr as an encapsulant. Nevertheless, we demonstrated that LaOBr can be used as an encapsulation dielectric and as a gate dielectric to control excitonic features in 2D materials, providing a high-κ playground for excitonic devices based on van der Waals heterostructures.

## Conclusions

We developed a straightforward and reproducible high-temperature flux growth method for producing large, stoichiometric crystals of the high-κ dielectric LaOBr. Through comprehensive characterization, we examined LaOBr in its crystal form, its cleaved (001) surface, and as an exfoliated flake, utilizing it as the insulating material in van der Waals heterostructure devices. With an out-of-plane static dielectric constant of 9, a high bandgap (5.3 eV), robust dielectric breakdown (8 MV cm^–1^) and low leakage currents (<10^–4^ A cm^–2^ for bulk flakes up to 1.5 MV/cm), LaOBr can be employed as a versatile high-k dielectric for gating and encapsulation of 2D materials.

Thanks to the quality of our LaOBr crystals and their ease of integration into van der Waals assembly processes, we successfully demonstrated field-effect transistor action on few-layer MoS_2_ with negligible hysteresis, low subthreshold slope (∼85 mV dec^–1^) and a low interfacial defect concentration (*D*_it_ ≃ 1.06 × 10^12^ cm^–2^ eV^–1^), with comparable performance with respect to nonlayered high-k dielectrics previously used for van der Waals integration.^7,8^ These results validate LaOBr as a layered, van der Waals-compatible, high-κ dielectric with a high bandgap and excellent breakdown voltage, representing an advancement toward the future scaling of electronics based on 2D materials.

Furthermore, our investigation showcased the utility of LaOBr as an encapsulating dielectric for studying gate-dependent excitonic features in van der Waals heterostructures, facilitating the exploration of excitonic devices with high-κ dielectric environments. These results could ignite further research on the control of excitonic ensembles in more complex van der Waals systems.^37^ As an example, in the case of excitonic properties of transition metal dichalcogenides (TMDCs), dipolar repulsive interactions in the transport of spatially indirect interlayer excitons are expected to be enhanced in high-κ dielectric environments, providing scaling possibilities for the long-range propagation of exciton ensembles.^38^

In the context of dielectric scaling over the past decades, LaOBr emerges as a van der Waals alternative to bulk oxides such as Al_2_O_3_, offering the critical advantage of its layered structure. Utilizing LaOBr as a high-κ wide-gap layered dielectric for 2D heterostructures lays the foundation for exploring electrical and optical devices within a high-κ dielectric environment, without any limitations on material combinations, thus exemplifying the paradigm of fully van der Waals integration.

## Methods

### Crystal Synthesis

As a starting material, lanthanum carbonate octahydrate (99.95%, Ganzhou Wanfeng Adv. Materials Tech. Co., Ltd.) was used without any further purification. LaBr_3_·7H_2_O was obtained by dissolving the oxide in concentrated hydrobromic acid (47%, p.a. grade, Fisher Scientific, Czech Republic) and crystallization of heptahydrate from acidic solution (pH ∼ 5) on a steam bath. Mixtures of 5 g of LaBr_3_·7H_2_O (made from lanthanum carbonate and hydrobromic acid), 4.2 g of NaBr (99%, p.a. grade, LachNer, Czech Republic), and 10.9 g of MgBr_2_ (98%, Sigma-Aldrich, Czech Republic) were placed in a corundum crucible. The crucible was initially heated to 150 °C for 5 h, where the majority of water slowly evaporates according to TGA. The furnace was placed in a well-ventilated area (fume hood) because of HBr gas production during this step. After dehydration, the mixture was slowly heated to 1000 °C at 2 °C min^–1^, the temperature was maintained for 48 h, and subsequently slow cooled down to 650 °C at 0.1 °C min^–1^, during which the growth of LaOBr takes place. After reaching 650 °C, the reaction was free-cooled to room temperature. The corundum crucible was leached in boiling water for 24 h to dissolve the salt flux, and the insoluble product was separated through vacuum filtration. The use of MgBr_2_ as a flux introduced a small amount of water-insoluble MgO which was removed by washing with dilute sulfuric acid (1:10, analytical reagent grade). The product was obtained as colorless platelets of LaOBr with lateral dimensions up to 1 mm.

### Bulk LaOBr Characterization

X-ray diffraction was carried out on a Bruker D8 Discover with Cu X-ray source (λ = 0.15418 nm, *U* = 40 kV, *I* = 40 mA). Diffractograms were collected in a range from 10° to 90° with a step of 0.02° and integration time of 0.2 s. The data was processed in HighScore plus software package, and the Rietveld refinement was conducted in Fullprof. Diffractograms were then normalized to the most intense peak. Characterization by Atomic Force Microscopy (AFM) was performed on NT-MDT Ntegra Spectra from NT-MDT in tapping mode and on an Asylum Research Cypher system. The morphology of the samples was investigated using scanning electron microscopy (SEM) with a FEG electron source (Tescan Lyra dual beam microscope). The samples were placed on carbon conductive tape. SEM measurements were carried out by using a 5 kV electron beam. The composition of the samples was determined using an energy dispersive spectroscopy (EDS) analyzer (X-MaxN) with a 20 mm^2^ SDD detector (Oxford Instruments). Data was evaluated using AZtecEnergy software. EDS measurements were carried out with a 15 kV acceleration voltage. Raman spectra were recorded with a WITec Confocal Raman Microscope (WITec alpha300 R, Ulm, Germany), equipped with a 532 nm laser and a spectrometer with a thermoelectrically cooled CCD camera sensor. The measurement was performed at room temperature with a 100× objective and a laser power of less than 1.2 mW to avoid sample degradation. The Raman modes were assigned according to DFT calculations in Quantum Espresso (Supporting Information, Note 4). ncAFM measurement were performed at a temperature of 5 K in a ultrahigh vacuum chamber (base pressure <10–11 mbar), suspended with 36 bungee cords for vibration insulation^39^ and equipped with a commercial Omicron qPlus low-temperature head. Imaging was achieved using stiff (*k* = 1800 N m^–1^) qPlus sensors with a high *Q* factor (*Q* = 10000–30000) and a resonance frequency in the kHz range (*f*_0_ = 25–45 kHz),^40^ that had a sharp W tip^41^ glued to the end of the oscillating prong. Deflection detection was achieved via a cryogenic preamplifier^42^ located in vacuum. Imaging conditions for the image in Figure 2e: bias applied to the sample *V*_S_ = +10 V, grounded tip, oscillation amplitude of *A* = 60 pm. LaOBr crystals were prepared for measurements by first ex-situ gluing them to a standard flag-type sample and later gluing a top post to the already fixed crystals. A conductive, two-component, silver epoxy glue suitable for UHV and low temperatures was used, and the curing was achieved by ambient annealing to 150 °C for 2 h. Prepared samples were introduced to UHV (base pressure <10^–10^ mbar) for in situ cleaving at room temperature, and quickly transferred to a measurement chamber with better pressure. Such cleaving methodology results in surfaces practically devoid of external adsorbates.^43,44^ Photoluminescence excitation (PLE) spectra were measured by a custom-made 5000 M spectrofluorometer (Horiba Jobin Yvon, Wildwood, MA, U.S.A.) using a steady state laser driven xenon lamp (Energetiq, a Hamamatsu Company) as the excitation sources. The detection part of the setup involved a single-grating monochromator and a TBX-04 photon-counting detector TBX-04 (Hamamatsu). The PLE spectra are corrected for the experimental distortion. Thermogravimetric experiments (TG) were performed on a Themys TGA (SETARAM instrument) with a heating rate of 10 °C min^–1^. The instrument was purged for at least three hours by the carrier gas.

### Device Fabrication

All devices used in this work were fabricated on top of SiO_2_(270 nm)/p^+^ Si substrates. For graphene field-effect structures, graphite (NGS) was exfoliated directly on the substrate, and graphene was identified by AFM. LaOBr was exfoliated on PDMS (gelpak) and transferred on graphene by a dry technique. In particular, LaOBr was picked up from PDMS with a polycarbonate (PC) membrane on a PDMS stamp. Then, LaOBr was transferred to the PC membrane on top of graphene. For MoS_2_ and MoSe_2_ field-effect structures, exfoliated few-layer graphite flakes were used as lateral electrodes. MoS_2_ and MoSe_2_ (2D Semiconductors) flakes were exfoliated on PDMS (gelpak). LaOBr was picked up from PDMS and then used to pick up TMDCs. The MoS_2_/LaOBr stack was then transferred on top of few-layer graphite flakes used as drains and sources by increasing the substrate temperature up to 170°C. The MoSe_2_/LaOBr stack was transferred similarly on top of a bottom Pt gate. The PC membranes were then cleaned by chloroform. All samples were annealed in high vacuum (10^–6^ mbar) at 340 °C for 6 h. Top gates and contacts on the MoS_2_/LaOBr device were all fabricated by e-beam lithography and Ti/Au (2/80 nm) evaporation and liftoff. The bottom gate in the MoSe_2_/LaOBr device and its electrodes were fabricated by e-beam lithography and metal evaporation of Pt (4nm) and Ti/Au (2/80 nm), respectively.

### Electrical Measurements

Transport measurements were carried out at room temperature under high vacuum (10^–6^ mbar) with dual-channel Keithley 2636 source measure units (SMUs). In all devices, a lateral contact was kept grounded, while the other lateral contact and the top gate were connected to the two channels of the same SMU. For dual-gate graphene field-effect structures, the silicon back-gate was connected to another Keithley 2636 SMU.

### Optical Measurements

Optical measurements were performed under vacuum in a He-flow cryostat at 4.6 K. Excitons in MoSe_2_ were excited with a confocal microscope by a continuous-wave 647 nm diode laser focused to the diffraction limit, and the emitted photons were collected through the same objective. The laser spot full-width at half-maximum measured approximately 1.2 μm. The emitted light was filtered by a 650 nm long-pass edge filter and then acquired using a spectrometer (Andor Shamrock) and recorded with a CCD (charge-coupled device) camera (Andor Newton).

## Data Availability

The data that support the findings of this study are available from the corresponding author on reasonable request.

## Supporting Information Available

(1) LaOBr synthesis approach according to Haeuseler et al. method (Figure S1). (2) Additional discussion of thermogravimetric analysis (Figure S2). (3) LaOBr crystals size and morphology (Figure S3). (4) Discussion of density Functional Theory for Raman mode identification. (5) Additional discussion of X-ray photoelectron spectroscopy (Figure S4). (6) Additional discussion of Photoluminescence measurements (Figure S5). (7) Comparison of LaOBr against other dielectrics. Additional figures: Surface quality and roughness of LaOBr (Figure S6). Graphene field-effect structure fabrication (Figure S7). Dual-gate measurements of a graphene field-effect structure B (Figure S8). Graphical comparison of dielectric constants (Figure S9). Breakdown field analysis of LaOBr (Figure S10). MoS_2_ transistor fabrication (Figure S11). Additional images of fabricated devices (Figure S12). Hysteresis in a MoS_2_ transistor–scan speed dependence (Figure S13). Temperature-dependent characteristics of a MoS_2_ transistor (Figure S14) (PDF)
