# Supplementary material for: High-κ Wide-Gap Layered Dielectric for Two-Dimensional van der Waals Heterostructures
Source: ACS Nano. 2024 Apr 1;18(15):10397–406. doi: 10.1021/acsnano.3c10411 (PMC11025129; doi:10.1021/acsnano.3c10411)
Supplement: Supplementary file 1 — nn3c10411_si_001.pdf [file nn3c10411_si_001.pdf]

Supplementary Information

for

A High-κ Wide-Gap Layered Dielectric For Two-Dimensional van der Waals Heterostructures

Aljoscha Söll[1§], Edoardo Lopriore[2,3§], Asmund Ottesen[2,3], Jan Luxa[1], Gabriele Pasquale[2,3], Jiri Sturala[1], František Hájek[4], Vítězslav Jarý[4], David Sedmidubský[1], Kseniia Mosina[1], Igor Sokolović[5,6], Saeed Rasouli[6], Tibor Grasser[5], Ulrike Diebold[6], Andras Kis[2,3], Zdeněk Sofer[1*]

[1]*Department of Inorganic Chemistry, University of Chemistry and Technology Prague, Technicka 5, 166 28 Prague 6, Czech Republic*
[2]*Institute of Electrical and Microengineering, École Polytechnique Fédérale de Lausanne (EPFL), CH-1015 Lausanne, Switzerland*
[3]*Institute of Materials Science and Engineering, École Polytechnique Fédérale de Lausanne (EPFL), CH-1015 Lausanne, Switzerland*
[4] *Institute of Physics of the Czech Academy of Sciences, v.v.i., Cukrovarnická 10, 162 00, Prague 6, Czech Republic*
[5] *Institute of Microelectornics, TU Wien, Gußhausstraße 27–29, 1040 Vienna, Austria*
[6] *Institute of Applied Physics, TU Wien, Wiedner Hauptstraße 8–10, 1040 Vienna, Austria*

§ *These authors contributed equally to this work.*

1. LaOBr synthesis of needles

We were able to reproduce the findings of Haeuseler *et al.* reported in Ref. [1]. In particular, we grew LaOBr from a mixture of La_2O_3 and anhydrous $LaBr_3$ with an excess of $LaBr_3$ as a flux inside a sealed quartz ampule, which produced a mixture of platelets and needles. The material was identified to be LaOBr by XRD (Figure S1a) and EDS (La:O:Br = 1:0.7:0.9). However, we found this method unsuitable for the synthesis LaOBr crystals with the purpose of exfoliation, since the majority of crystals form as needles growing along the <001> direction. Additionally, this method consumes a large amount of anhydrous $LaBr_3$ and presents potential hazards. In fact, if the $LaBr_3$ contains residual water, it will lead to the formation of gaseous HBr upon heating which could cause the ampule to explode.

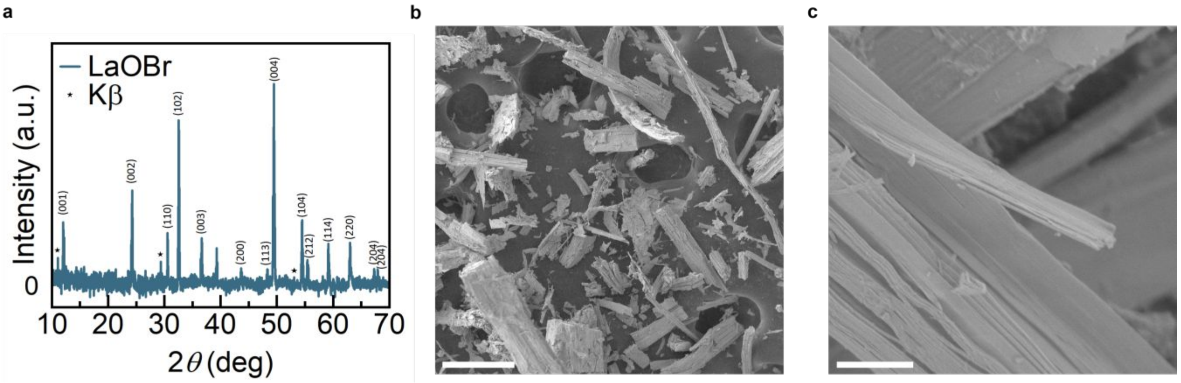

Figure S1. a, X-Ray diffractogram of LaOBr needles from Haeuseler *et al.* method[1]. **b,** Scanning electron microscopy (SEM) image of LaOBr needles produced via this synthesis. Scale bar 200 μm. **c,** Magnified SEM image of LaOBr needle. Scale bar: 5 μm.

2. Thermogravimetric analysis

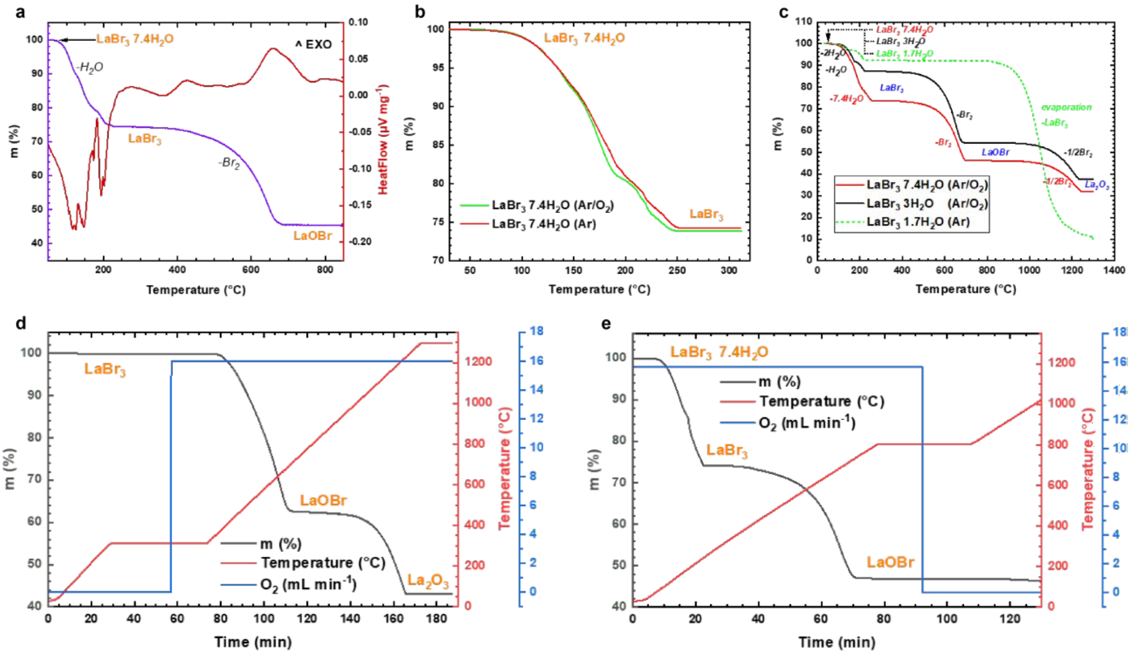

Figure S2. a, TGA analysis of LaBr$_3$ · 7.4 H$_2$O. **b,** TGA of the initial dehydration step in different atmospheres (Ar vs. Ar/O$_2$). **c,** TGA of LaBr$_3$ with different levels of hydration in Ar and. Ar/O$_2$ atmospheres. **d**, LaBr3 · 7.4 H$_2$O TGA in Ar-atmosphere which is switched to Ar/O$_2$ at 350 °C. **e**, LaBr$_3$ · 7.4 H$_2$O TGA in Ar/O$_2$-atmosphere which is switched to Ar at 800 °C.

We conducted thermogravimetric analysis (TGA) coupled with differential scanning calorimetry (DSC) on a sample of LaBr$_3$ · 7.4H$_2$O (Figure S2a) in a dynamic Ar/O$_2$ atmosphere (64/16 mL min^{-1}). The data indicates that the evaporation of water starts about 70 °C according to Eq. (1) until a temperature of 230 °C is reached, corresponding to a mass loss of 25.6% (theoretical mass loss 26%). According to DSC, the evaporation of water is endothermic.

$$\mathrm{LaBr_3 \cdot 7.4H_2O\ (s) \rightarrow LaBr_3\ (s)\ +\ 7.4\ H_2O\ (g)} \qquad (1)$$

The next decomposition (Eq. 2) starts at approximately 350 °C, and corresponds to the formation of LaOBr by oxidation of bromide anions to bromine. The decomposition is finished

at about 710 °C and resulted in a mass loss of 29% with respect to the initial mass, which is in good agreement with the theoretical value (28.1%). This oxidation is exothermic.

$$LaBr_3\ (s) \rightarrow LaOBr\ (s) + Br_2\ (g) \tag{2}$$

Previous works[2] have suggested hydrolysis as the mechanism for the formation of LaOBr, as in Eq. 3:

$$LaBr_3(s) + H_2O\ (l) \rightarrow LaOBr(s) + 2\ HBr(g) \tag{3}$$

However, in our case, we propose the oxidation in dynamic atmosphere with atmospheric oxygen as the driving factor in the formation of our LaOBr crystals, following Eq. 4:

$$LaBr_3(s) + \tfrac{1}{2}\ O_2\ (g) \rightarrow LaOBr(s) + Br_2(g) \tag{4}$$

To distinguish between these two mechanisms, we have conducted the TG experiments in an inert atmosphere (Ar; 160 mL min^{-1}) and in an environment containing oxygen (Ar/O_2 = 64/16 mL min^{-1}) (Figure S2b). In both cases, the mass loss of the first step was almost the same (25.8% in Ar, 26.1% in Ar/O_2). The small difference in the mass loss was attributed to the slightly variable content of water in the initial material. If the TG experiment was performed with an initial vacuum step at 30 °C, a certain portion of water was already removed. Therefore, the composition had to be corrected based on our observation that the initial loss corresponds to the evaporation of water only. Regardless ofthe amount of crystal water, $LaBr_3$ was formed followed by subsequent oxidation of bromide to bromine and formation of LaOBr. In case that the obtained LaOBr was further heated in an oxygen containing atmosphere (case of Ar/O_2 environment; 64/16 mL min^{-1}), we observed its further oxidtion to La_2O_3, which started at about 900 °C, according to Eq 5. The mass loss of 14.1 % (for $7.4H_2O$) during this step is in excellent agreement with the theoretical value of 14 % (Figure S2c, red and black line).

$$2LaOBr\ (s) + \frac{1}{2}O_2 \rightarrow La_2O_3\ (s) + Br_2\ (g) \tag{5}$$

However, in case of an inert (Ar; 200 mL min^{-1}) environment, we observed the formation of $LaBr_3$ and its evaporation soon after its melting point (785 °C)[3] without formation of LaOBr (Figure S2c).

To further clarify the reason why the O_2 environment is a key element for the formation of LaOBr, we performed an additional TGA analysis as reported in Figure S2d-e. In the case of Figure S1d, $LaBr_3$ was heated in an Ar atmosphere (160 mL min^{-1}) up to 300 °C, and no mass loss was observed. Then, the atmosphere was changed to Ar/O_2 (144/16 mL min^{-1}). At 350 °C, we observed the start of the formation of LaOBr, followed by the subsequent formation of La_2O_3 (Figure S1d). On the other hand, if the hydrate was heated in an Ar/O_2 atmosphere (144/16 mL min^{-1}), as in Figure S2e, LaOBr was finally formed. Further heating of LaOBr in Ar atmosphere (160 mL min^{-1}) only up to 1000 °C did not cause any significant change to the material (Figure S1e).

3. Crystal size and morphology

Crystal growth with our method (see main text and Methods) produced LaOBr crystals as thin platelets with varying sizes up to 1 mm. Some corners appear slightly rounded due to the minor dissolution of LaOBr during the separation and cleaning steps (Figure S3).

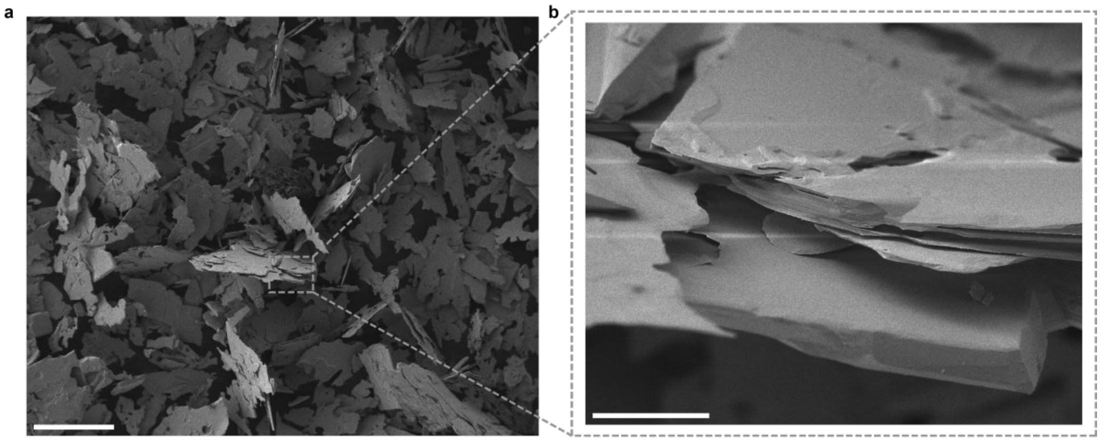

Figure S3. a, SEM image of LaOBr crystals in the form of thin platelets. Scale bar: 200 μm. **b,** Magnified image on the edge of a single platelet with a thickness of ~10 μm. Scale bar: 20 μm.

4. Density functional theory for Raman modes

The calculations were performed using density functional theory (DFT) in Quantum Espresso.[4] The calculations used PBEsol[5] or vdW-DF3-opt1[6] functionals, with the norm-conserving (NC) or projector-augmented wave methods (PAW). The initial crystal structure of LaOBr (consisting of two La, Br and O atoms) was obtained from Ref. [7]. The cell was optimized to a minimum using a K mesh of 4×4×4 and a $E_{cut} = 250\ Ry$, followed by structure relaxation using a K mesh 6×6×6 and plane wave cut-off energy $E_{cut} = 500\ Ry$. This structure was used for the calculation of the theoretical Raman (and IR) spectra to help with the assignment of Raman modes (Figure 1e in the main text).

The primitive cell of LaOBr is composed of six unique atoms in its structure (two formula units) and belongs to the P4/nmm space group (tetragonal PbFCl-type lattice). Therefore, there are 18 vibrational modes:

$$\Gamma = 3E_g + 1B_{1g} + 2A_{1g} + 3E_u + 3A_{2u} \quad (5)$$

In particular, the E_u and A_{2u} modes are IR active, since two of six modes are acoustic modes. Instead, A_{1g},$B_{1g}$and E_g are Raman active. The DFT results are summarized in Table S1.

DFT	Pseudopotential	E_{g1}	A_{1g1}	E_{g2}	A_{1g2}	B_{1g}	E_{g3}
VDW-DF3-OPT1	NC	108.5	124.9	133.8	186.5	315.8	412.6
VDW-DF3-OPT1	PAW	123.1	137.1	139.2	188.2	318.7	412.0
PBEsol	PAW	115.9	136.1	141.1	188.3	317.5	411.3
Experimental		109.5	127.3	132.3	188.5	329.0	427.1

Table S1. DFT results obtained as described in Supplementary Note 4.

5. X-ray photoelectron spectroscopy

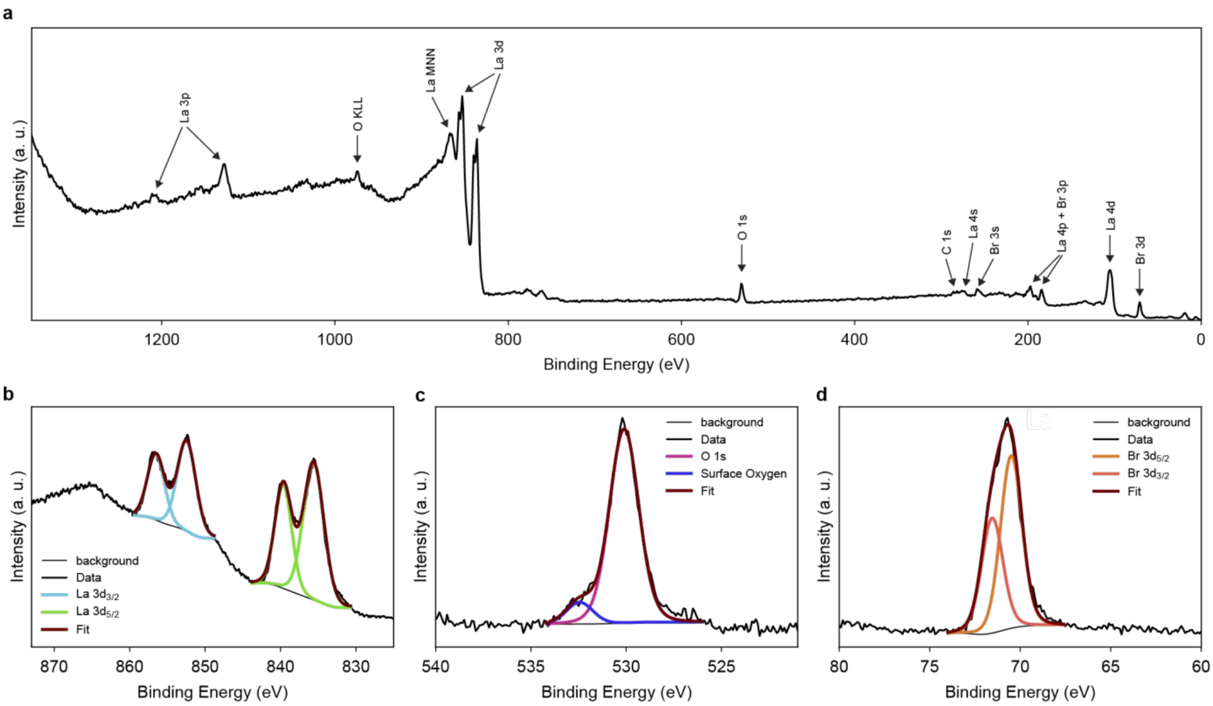

Figure S4. a, La 3d XPS spectrum. **b,** O 1s XPS spectrum. **c,** Br 3d XPS Spectrum. **d,** Survey Spectrum.

The survey spectrum shown in Figure S4a shows the absence of surface impurities, as only La, Br, and O are present. Furthermore, the intensity of C 1s from adventitious contamination is very low, suggesting a clean surface of LaOBr. The La 3d spectrum in Figure S4b comprises doublet located at 835.5 eV and 852.5 eV, originating from La 3d5/2 and La 3d3/2, respectively. The La 3d components are further split into multiplets with the second component located at 839.8 eV for La 3d5/2 and 856.5 eV for La 3d3/2 . The multiplet splitting of 4.0 - 4.3 eV is narrower than that previously reported for La_2O_3 (4.6 eV),[8] but larger than that of $LaBr_3$ (3.7 eV),[9] possibly due to presence of both La-O and La-Br bonds within the structure. The structure of the spectrum and the presence of multiplets is believed to be caused by a shake-up process where electrons from O 2p band are transferred to an empty La 4f orbitals.[10] Other effects such as hybridization between La 4f and O 2p orbitals may also contribute to the observed phenomenon.[11] The O 1s spectrum in Figure S4c shows two features located at 529.9 and 532.4 eV. The high intensity peak at 529.9 eV is associated with La-O bond, well in agreement with previous reports, while the second peak can be ascribed to adventitious oxygen contamination.[11] Finally, the Br 3d spectrum in Figure S4d reports a doublet composed of two spin components at 70.4 (Br $3d_{5/2}$) and 71.4 eV (Br $3d_{3/2}$), indicating a single oxidation state of Br in LaOBr.

6. Photoluminescence excitation measurements

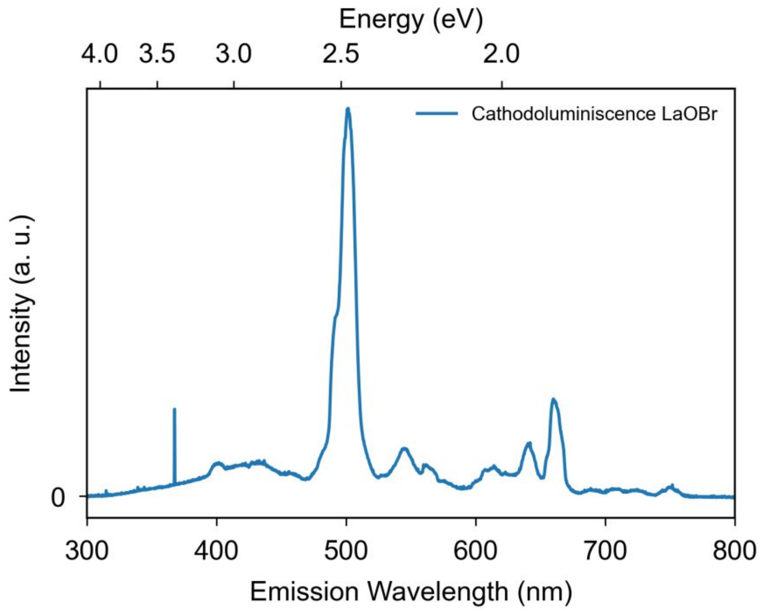

Figure S5. Cathodoluminescence spectrum of a bulk LaOBr crystal with emission wavelength at 501 nm (4f-4f transition of a praseodymium impurity).

The photoluminescence excitation (PLE) spectra of our LaOBr crystals are shown in Figure 2f in the main text. The emission wavelength is set at 501 nm (2.47 eV), corresponding to the *4f-4f* transition of a praseodymium impurity.[12] A cathodoluminescence spectrum of LaOBr is shown in Figure S5 to show the praseodymium impurity transitions. To estimate the bandgap of LaOBr from our PLE signal, we applied a density of states (DOS) model as described in Ref. [13]. The model is applicable with assumption of direct bandgap of the material which is fulfilled in the case of LaOBr.[14] Such a model gives an estimate of the bandgap energy of 5.30 eV and a characteristic Urbach energy of 0.39 eV. We note that the obtained bandgap is comparable to that reported in previous works by diffuse reflectance measurements (5.38 eV).[15] Furthermore, the high value of the characteristic Urbach tail energy suggests that several distinct mechanisms, like crystal disorder, impurities or phonon-related absorption, contribute

to sub-bandgap absorption. Following the model in Ref. [13], we obtained a fitting parameter $\beta \approx 1$, which indicates that localized states are the dominant origin for the sub-bandgap absorption rather than electrostatic fluctuations or the Franz-Keldysh effect. Moreover, a distinct peak at 5.12 eV is observed, probably related to an exciton bound on an unknown defect. All the above-mentioned effects make the exact bandgap value determination challenging. Nonetheless, our analysis reveals a gap that is lying above 5 eV in energy, thus making LaOBr a wide-gap high-k dielectric.

We note that the PLE spectra in Figure 2f follow the intrinsic absorption spectrum till the high absorption above bandgap occurs. Such high absorption leads to the excitation of a region with limited depth, causing the PLE signal to drop, as previously reported for PLE measurements on hBN.[16] This explains the incongruence at energies higher than 5.5 eV with respect to the ideal monotonic model reported in Ref. [13].

7. Benchmarking LaOBr against other 2D dielectrics

Material	Exfoliation	κ	E_G	SS	I_{ON}/I_{OFF}	Leakage
LaOBr	*Yes*	9	5.3	85	10^8	$<10^{-4}$
hBN [17,18]	*Yes*	5	6	69	10^8	$<10^{-4}$-10^{-1}*
Mica [19]	*Yes*	8.1	10.5	72	10^8	-
Bi_2SeO_5 [20]	*Yes*	16	3.6	70	10^8	$<10^{-6}$
STO [21,22]	*No*	17-20	3.3	66	10^8	$<10^{-2}$
BTO [23]	*No*	4700	3.3	-	10^5-10^7	-
LaOCl [24]	*No*	-	5.9^{theo}	77	10^7	-
Sb_2O_3 [25]	*No*	11.5	4	64	10^8	10^{-7}

^{theo} Value taken from theoretical calculations. *No exact value given; value estimated from graph.

Table S1

To emphasize the potential of LaOBr as a layered dielectric, we assembled Table S1 showing the properties of known 2D dielectrics. Here, we compare material-specific parameters such as bandgap and dielectric constant, as well as characteristic figures of merit when the material is used in a TMDC based FET. Additionally, for each material, we indicate whether it has been demonstrated to be exfoliable from its bulk.

Some materials show exceptionally high dielectric constants (Bi_2SeO_5, STO, BTO), but are limited by their small bandgaps (3.6 eV or less). Other materials (LaOCl, Sb_2O_3) show promising properties, but have so far only been accessible through thin film growth methods, which makes them less accessible for creating high-quality van der Waals heterostructures. Instead, the dielectric integration of mica with 2D materials is hindered by the inherent hydrophilicity of its surface.[26,27] The hydroxyl-terminated surface has been reported to attract a strongly bound water adlayer leading to a large hysteresis in graphene channel devices, due to strong and persistent hole doping.[28]

Out of all these materials, LaOBr suffers from no major shortcomings. It can be exfoliated easily, features a high dielectric constant and bandgap, and shows overall good device performance.

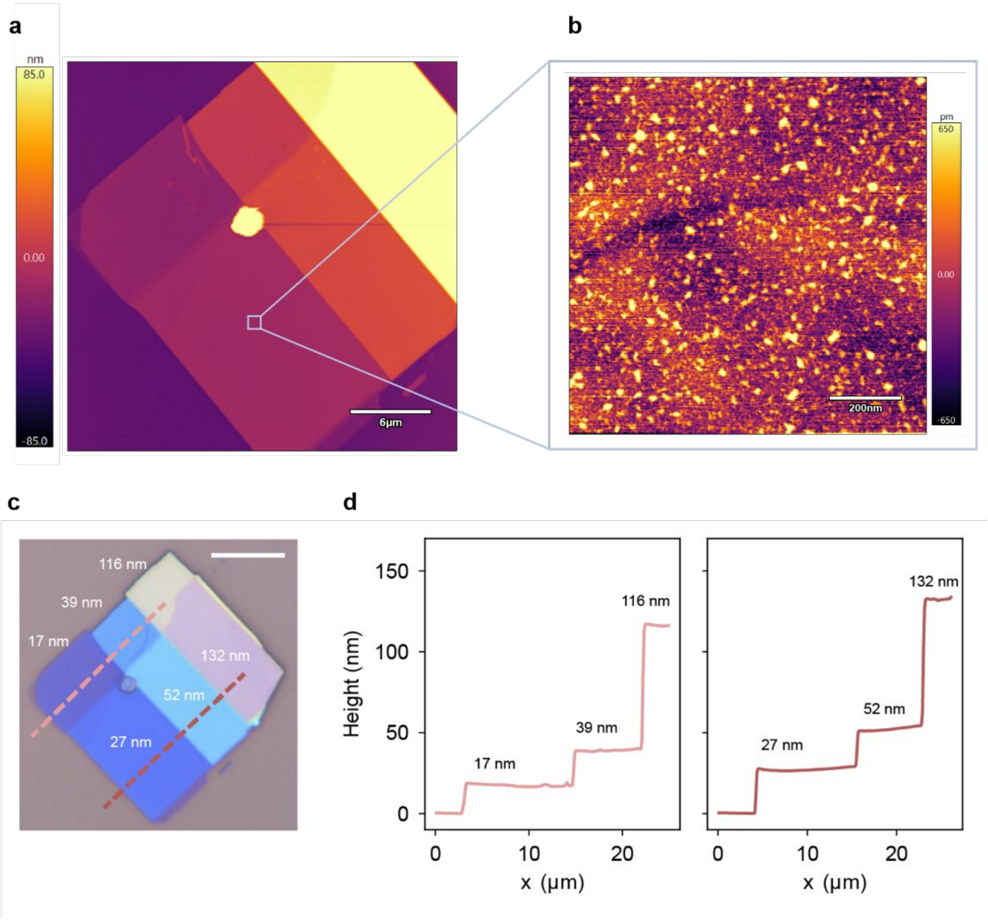

Figure S6. LaOBr stairs and roughness a, LaOBr flake with stairs of different heights, as reported in Figure 2 in the main text. Scale bar: 6 μm. **b,** Roughness scan of a flat LaOBr region highlighted in (a). We measure a standard deviation of 254.5 pm and an average deviation of 190.4 pm in height, indicating a smooth surface comparable with other exfoliated high-quality 2D materials as hBN.[29,30] **c,** Flake of LaOBr on a SiO_2 substrate (270 nm) as in Figure 2d in the main text. Two shaded lines in light red and dark red represent two height measurements obtained by AFM. Scale bar: 10 μm. **e,** Height measurements of the flakes in (c) obtained by AFM, with colors corresponding to the light red and dark red shaded lines in (c).

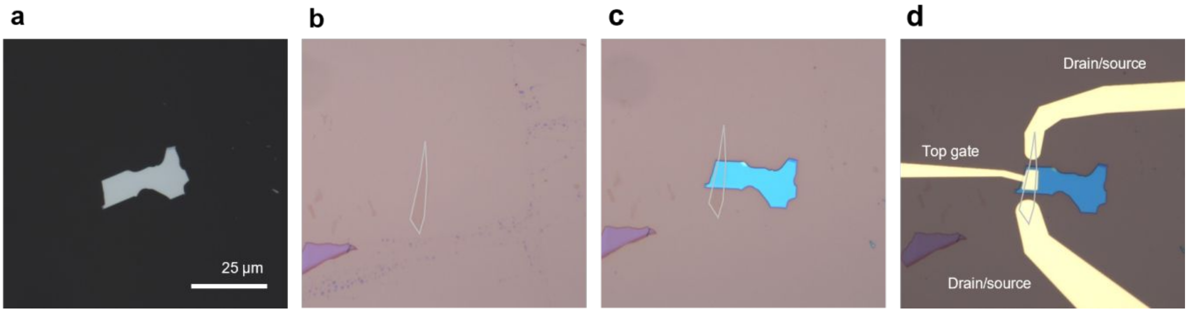

Figure S7. Graphene field-effect structure fabrication (device A). a, LaOBr flake exfoliated on a PDMS (gelpak) substrate. Scale bar: $25\ \mu m$. **b,** Graphene monolayer flake (grey) as exfoliated on a SiO_2 substrate. **c,** The LaOBr flake is picked up by a PC stamp and transferred over the graphene layer, as described in the Methods section. **d,** Ti/Au lines are evaporated to form drain/source and top gate electrodes, giving the graphene field-effect structure as reported in Figure 3 in the main text.

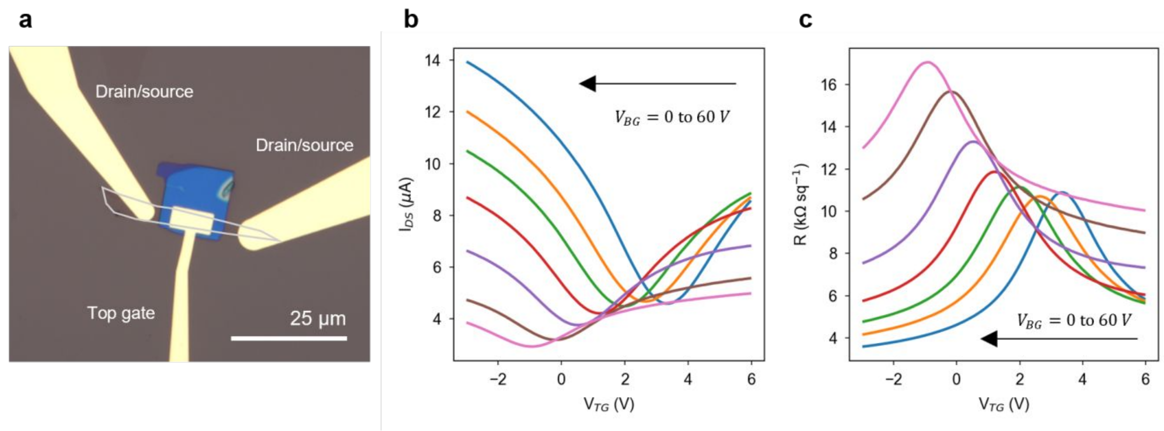

Figure S8. Dual-gate measurements of graphene field-effect device B. a, Optical micrograph of the graphene field-effect device B, with the graphene layer highlighted in grey. The role of the Ti/Au electrodes is labeled. Scale bar: $25\ \mu\mathrm{m}$. **b-c,** Drain-source current I_{DS} (b) and resistance R (c) as a function of the applied top gate voltage V_{TG} for different fixed values of V_{BG}. The range of bottom gate voltages is swept between 0 V and 60 V. The obtained Dirac point shift is reported in Figure 3 in the main text.

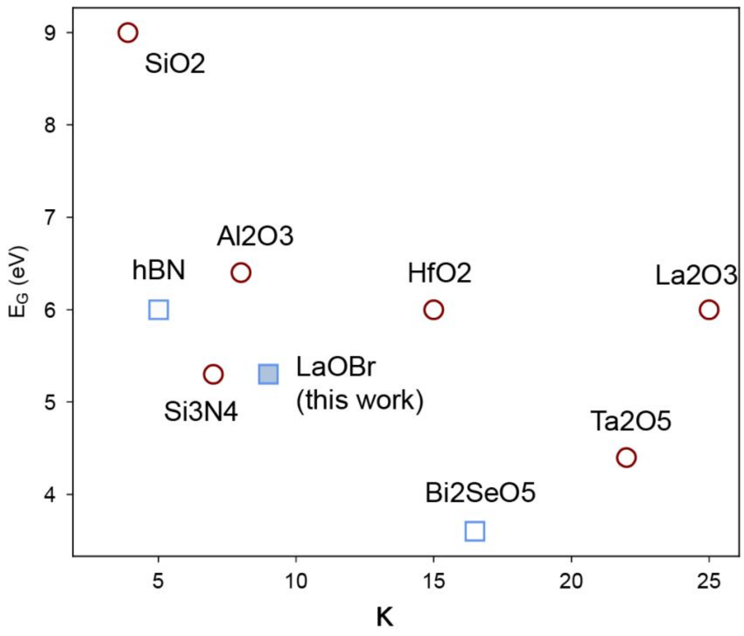

Figure S9. Static dielectric constant and bandgap of dielectrics. The three exfoliable layered dielectrics up-to-date (hBN,[31,32] Bi_2SeO_5,[20] and our LaOBr crystals) are compared with 3D bulk oxides:[33] In particular, we focused on bulk materials that are commonly used in combination with 2D semiconductors in electronic and photonic devices. Blue squares and red circles indicate 2D and 3D dielectrics, respectively. LaOBr is characterized by a bandgap close to that of Si_3N_4 (5.3 eV), and a static dielectric constant that is slightly higher than that of ALD-Al_2O_3 ($\kappa \sim 8$).

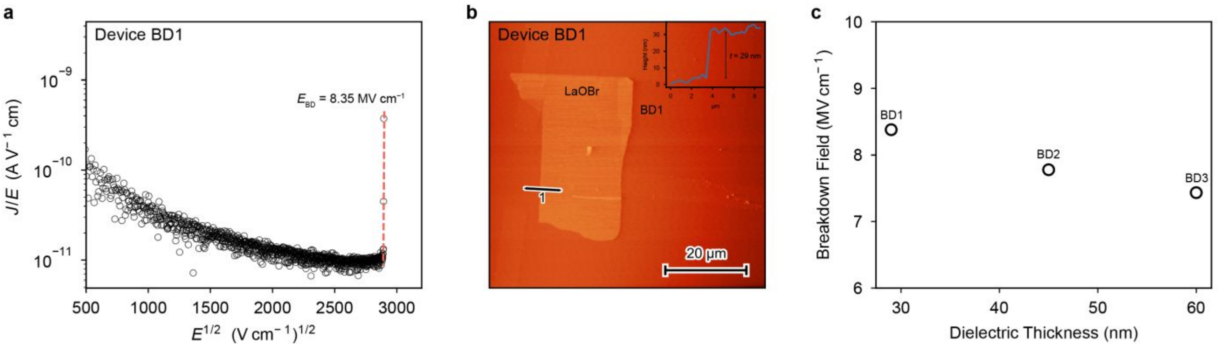

Figure S10. Breakdown field of bulk LaOBr. a, Frenkel-Poole plot of Breakdown Device BD1. Breakdown appears as sudden linear increase of J/E at a field of $E_{\text{BD}} = 8.35\ \text{MV cm}^{-1}$. **b**, AFM image of BD1 showing the LaOBr flake on top of a gold electrode. The dielectric thickness was measured as $t = 29$ nm. **c**, The breakdown field values for three devices are plotted in relation to their dielectric thickness. Here, the average breakdown field was determined as $E_{BD} = 7.85 \pm 0.47\ \text{MV cm}^{-1}$. We note that this value works as an approximate estimate of the breakdown field in LaOBr. Furthermore, when comparing the individual values for all BD devices, we observe a trend of decreasing breakdown field with increasing dielectric thickness. This behavior aligns well with previous observations reported for materials like hBN and SiO_2.[34] The underlying reason for this behavior is attributed to an intensified local electric field in thicker dielectrics for the same applied voltage, due to the contributions of both the applied field and the material polarization.[35]

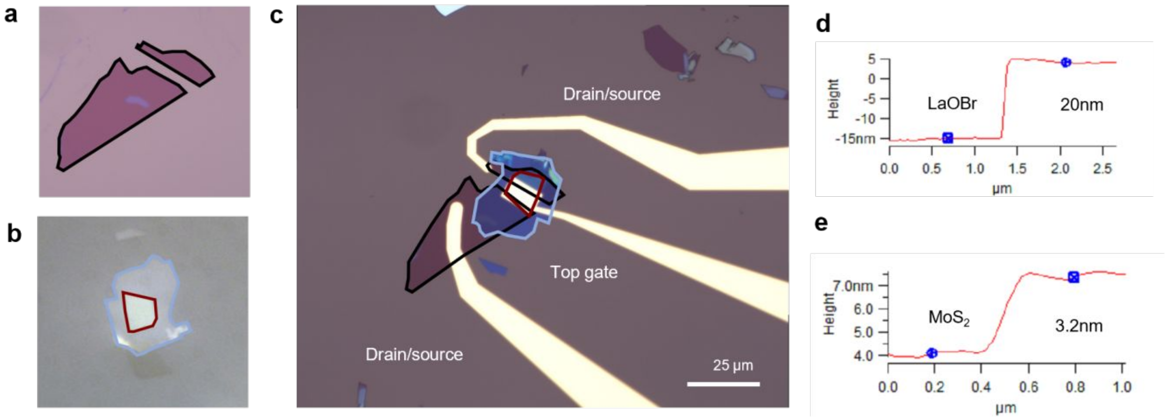

Figure S11. MoS_2-LaOBr transistor fabrication. a, Exfoliated flakes of few-layer graphite on a SiO_2 substrate, highlighted by black contour lines. **b,** As described in the Methods section in the main text, flakes are picked up successively by a PC stamp over a PDMS substrate. Here, the optical micrograph shows the LaOBr flake (blue), the first material to be picked up by the PC stamp, and the MoS_2 flake (red), successively picked up by LaOBr by van der Waals bonds. Then, the stamp is transferred on the graphene flakes in (a) in order to form a laterally contacted MoS_2 layer with LaOBr as top dielectric. **c,** The evaporated Ti/Au drain/source and top gate electrodes complete the MoS_2 field-effect transistor with LaOBr as gate dielectric. **d-e,** AFM height measurements of the gate LaOBr dielectric (20 nm) and the 4L-MoS_2 flake (3.2 nm).

a b c d

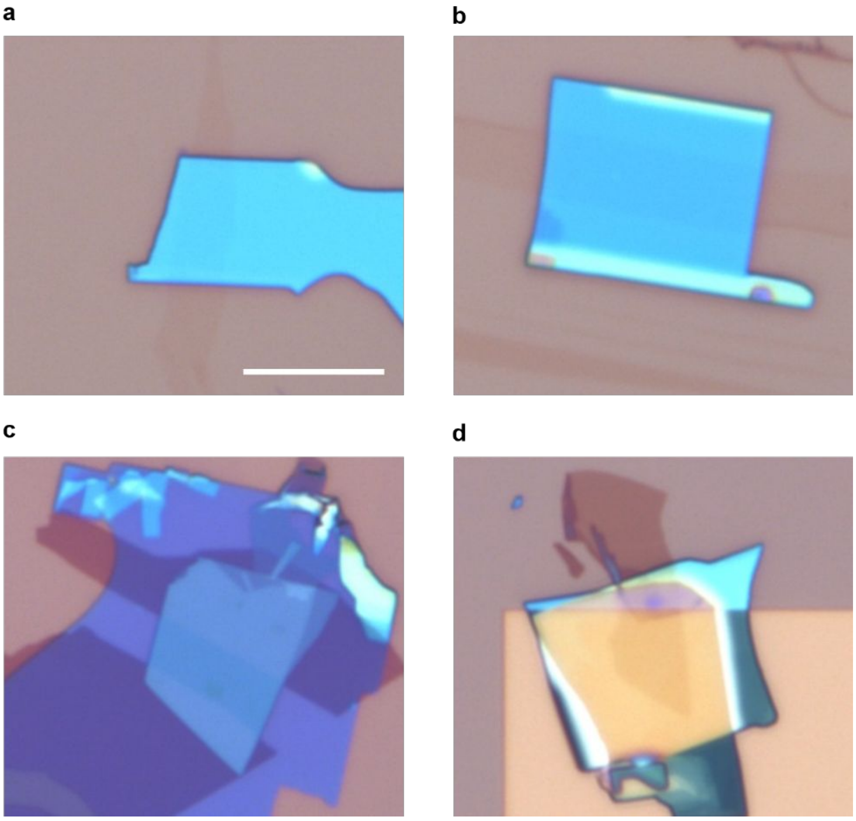

Figure S12. Device images. a-d, Optical micrographs of the graphene field-effect devices (a,b), the MoS_2 field-effect transistor (c) and the $MoSe_2$ gated device (d) prior to the top-gate lithography step. The images showcase high-quality interfaces with morphological uniformity. Scale bar: $10\ \mu m$.

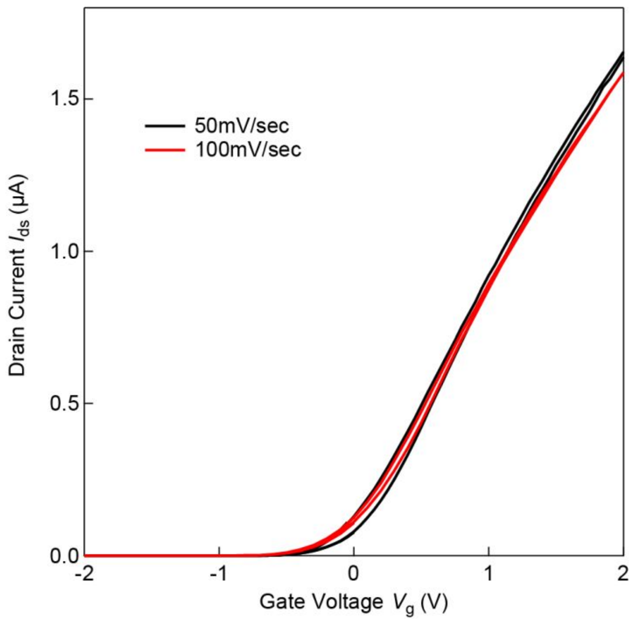

Figure S13. Hysteresis with different scan speeds. Gate sweeps of a MoS_2 transistor at fixed bias ($V_{DS} = 20\ \mathrm{mV}$) and different scan speeds of 50 mV/s (black) and 100 mV/s (red). The two curves show comparable hysteresis, with a wider window for the slower scan.

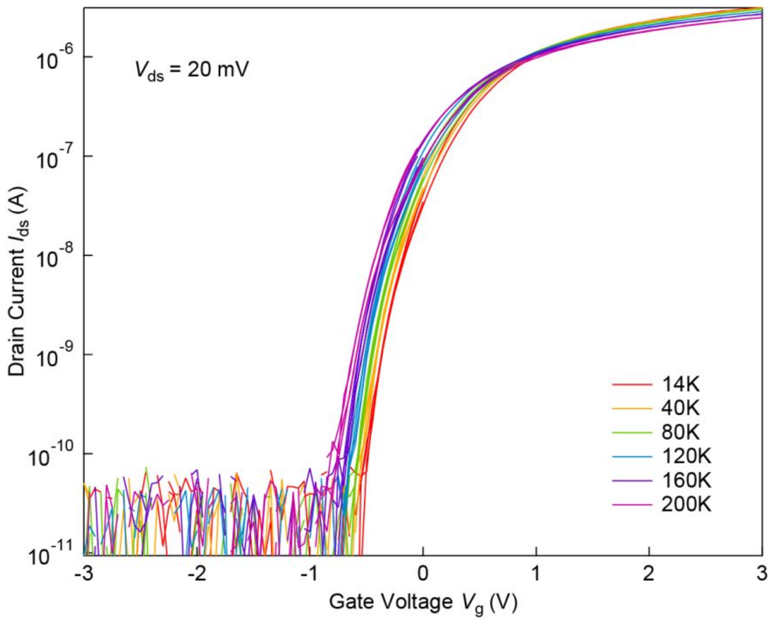

Figure S14. Temperature-dependent MoS_2 characteristics. Gate sweeps of a MoS_2 transistor at fixed bias ($V_{DS} = 20\ \mathrm{mV}$) and different temperatures in logarit. The subthreshold swing of all curves is comparable, thus confirming the low density of interfacial defective states D_{it}, as estimated in the main text. In agreement with this, the measured hysteresis is comparable independently on temperature, with maximum values always in the range of 100 mV. These observations confirm that high-quality van der Waals interfaces are obtained between MoS_2 and LaOBr in our device.

References

(1) Haeuseler, H.; Jung, M. Single Crystal Growth and Structure of LaOBr and SmOBr. *Mater. Res. Bull.* **1986**, *21* (11), 1291–1294. https://doi.org/10.1016/0025-5408(86)90062-0.

(2) Zhang, T.; Li, H.; Zhao, C.; Yu, J.; Hu, Y.; Cui, L.; He, H. Preparation of Anhydrous Lanthanum Bromide for Scintillation Crystal Growth. *J. Rare Earths* **2012**, *30* (12), 1245–1248. https://doi.org/10.1016/S1002-0721(12)60214-2.

(3) Rycerz, L.; Gaune-Escard, M. Enthalpy of Phase Transitions and Heat Capacity of Stoichiometric Compounds in LaBr3-MBr Systems (M=K, Rb, Cs). *J. Therm. Anal. Calorim.* **1999**, *56* (1), 355–363. https://doi.org/10.1023/A:1010102802661.

(4) Giannozzi, P.; Baroni, S.; Bonini, N.; Calandra, M.; Car, R.; Cavazzoni, C.; Ceresoli, D.; Chiarotti, G. L.; Cococcioni, M.; Dabo, I.; Dal Corso, A.; de Gironcoli, S.; Fabris, S.; Fratesi, G.; Gebauer, R.; Gerstmann, U.; Gougoussis, C.; Kokalj, A.; Lazzeri, M.; Martin-Samos, L.; Marzari, N.; Mauri, F.; Mazzarello, R.; Paolini, S.; Pasquarello, A.; Paulatto, L.; Sbraccia, C.; Scandolo, S.; Sclauzero, G.; Seitsonen, A. P.; Smogunov, A.; Umari, P.; Wentzcovitch, R. M. QUANTUM ESPRESSO: A Modular and Open-Source Software Project for Quantum Simulations of Materials. *J. Phys. Condens. Matter* **2009**, *21* (39), 395502. https://doi.org/10.1088/0953-8984/21/39/395502.

(5) Perdew, J. P.; Ruzsinszky, A.; Csonka, G. I.; Vydrov, O. A.; Scuseria, G. E.; Constantin, L. A.; Zhou, X.; Burke, K. Restoring the Density-Gradient Expansion for Exchange in Solids and Surfaces. *Phys. Rev. Lett.* **2008**, *100* (13), 136406. https://doi.org/10.1103/PhysRevLett.100.136406.

(6) Chakraborty, D.; Berland, K.; Thonhauser, T. Next-Generation Nonlocal van Der Waals Density Functional. *J. Chem. Theory Comput.* **2020**, *16* (9), 5893–5911. https://doi.org/10.1021/acs.jctc.0c00471.

(7) Krämer, K.; Schleid, T.; Schulze, M.; Urland, W.; Meyer, G. Three Bromides of Lanthanum: LaBr2, La2Br5, and LaBr3. *Z. Für Anorg. Allg. Chem.* **1989**, *575* (1), 61–70. https://doi.org/10.1002/zaac.19895750109.

(8) Sunding, M. F.; Hadidi, K.; Diplas, S.; Løvvik, O. M.; Norby, T. E.; Gunnæs, A. E. XPS Characterisation of in Situ Treated Lanthanum Oxide and Hydroxide Using Tailored Charge Referencing and Peak Fitting Procedures. *J. Electron Spectrosc. Relat. Phenom.* **2011**, *184* (7), 399–409. https://doi.org/10.1016/j.elspec.2011.04.002.

(9) Suzuki Takehiko Ishii, S.; Sagawa, T. X-Ray Photoemission Spectra of 4d and 3d Electrons in Lanthanum- and Cerium-Halides. *J. Phys. Soc. Jpn.* **1974**, *37* (5), 1334–1340. https://doi.org/10.1143/JPSJ.37.1334.

(10) Burroughs, P.; Hamnett, A.; Orchard, A. F.; Thornton, G. Satellite Structure in the X-Ray Photoelectron Spectra of Some Binary and Mixed Oxides of Lanthanum and Cerium. *J. Chem. Soc. Dalton Trans.* **1976**, No. 17, 1686–1698. https://doi.org/10.1039/DT9760001686.

(11) Li, J. P. H.; Zhou, X.; Pang, Y.; Zhu, L.; Vovk, E. I.; Cong, L.; Bavel, A. P. van; Li, S.; Yang, Y. Understanding of Binding Energy Calibration in XPS of Lanthanum Oxide by in Situ Treatment. *Phys. Chem. Chem. Phys.* **2019**, *21* (40), 22351–22358. https://doi.org/10.1039/C9CP04187G.

(12) Li, Y.-C.; Chang, Y.-H.; Lin, Y.-F.; Chang, Y.-S.; Lin, Y.-J. Luminescent Properties of Trivalent Praseodymium-Doped Lanthanum Aluminum Germanate LaAlGe2O7. *J. Phys. Chem. Solids* **2007**, *68* (10), 1940–1945. https://doi.org/10.1016/j.jpcs.2007.06.013.

(13) Bleuse, J.; Perret, S.; Curé, Y.; Grenet, L.; André, R.; Mariette, H. Optical Determination of the Band Gap and Band Tail of Epitaxial ${\mathrm{Ag}}_{2}\mathrm{Zn}\mathrm{Sn}{\mathrm{Se}}_{4}$ at Low Temperature. *Phys. Rev. B* **2020**, *102* (19), 195205. https://doi.org/10.1103/PhysRevB.102.195205.

(14) Shwetha, G.; Kanchana, V.; Yedukondalu, N.; Vaitheeswaran, G. Ab Initio Study of Scintillating Lanthanide Oxyhalide Host Materials. *Mater. Res. Express* **2015**, *2* (10), 105901. https://doi.org/10.1088/2053-1591/2/10/105901.

(15) Kim, D.; Park, S.; Kim, S.; Kang, S.-G.; Park, J.-C. Blue-Emitting Eu2+-Activated LaOX (X = Cl, Br, and I) Materials: Crystal Field Effect. *Inorg. Chem.* **2014**, *53* (22), 11966–11973. https://doi.org/10.1021/ic5015576.

(16) Museur, L.; Feldbach, E.; Kanaev, A. Defect-Related Photoluminescence of Hexagonal Boron Nitride. *Phys. Rev. B* **2008**, *78* (15), 155204. https://doi.org/10.1103/PhysRevB.78.155204.
(17) Vu, Q. A.; Fan, S.; Lee, S. H.; Joo, M.-K.; Yu, W. J.; Lee, Y. H. Near-Zero Hysteresis and near-Ideal Subthreshold Swing in h-BN Encapsulated Single-Layer MoS2 Field-Effect Transistors. *2D Mater.* **2018**, *5* (3), 031001. https://doi.org/10.1088/2053-1583/aab672.
(18) Knobloch, T.; Illarionov, Y. Y.; Ducry, F.; Schleich, C.; Wachter, S.; Watanabe, K.; Taniguchi, T.; Mueller, T.; Waltl, M.; Lanza, M.; Vexler, M. I.; Luisier, M.; Grasser, T. The Performance Limits of Hexagonal Boron Nitride as an Insulator for Scaled CMOS Devices Based on Two-Dimensional Materials. *Nat. Electron.* **2021**, *4* (2), 98–108. https://doi.org/10.1038/s41928-020-00529-x.
(19) Zou, X.; Xu, J.; Liu, L.; Wang, H.; Lai, P.-T.; Tang, W. M. Damage-Free Mica/MoS2 Interface for High-Performance Multilayer MoS2 Field-Effect Transistors. *Nanotechnology* **2019**, *30* (34), 345204. https://doi.org/10.1088/1361-6528/ab1ff3.
(20) Zhang, C.; Tu, T.; Wang, J.; Zhu, Y.; Tan, C.; Chen, L.; Wu, M.; Zhu, R.; Liu, Y.; Fu, H.; Yu, J.; Zhang, Y.; Cong, X.; Zhou, X.; Zhao, J.; Li, T.; Liao, Z.; Wu, X.; Lai, K.; Yan, B.; Gao, P.; Huang, Q.; Xu, H.; Hu, H.; Liu, H.; Yin, J.; Peng, H. Single-Crystalline van Der Waals Layered Dielectric with High Dielectric Constant. *Nat. Mater.* **2023**, 1–6. https://doi.org/10.1038/s41563-023-01502-7.
(21) Yang, A. J.; Han, K.; Huang, K.; Ye, C.; Wen, W.; Zhu, R.; Zhu, R.; Xu, J.; Yu, T.; Gao, P.; Xiong, Q.; Renshaw Wang, X. Van Der Waals Integration of High-κ Perovskite Oxides and Two-Dimensional Semiconductors. *Nat. Electron.* **2022**, *5* (4), 233–240. https://doi.org/10.1038/s41928-022-00753-7.
(22) Huang, J.-K.; Wan, Y.; Shi, J.; Zhang, J.; Wang, Z.; Wang, W.; Yang, N.; Liu, Y.; Lin, C.-H.; Guan, X.; Hu, L.; Yang, Z.-L.; Huang, B.-C.; Chiu, Y.-P.; Yang, J.; Tung, V.; Wang, D.; Kalantar-Zadeh, K.; Wu, T.; Zu, X.; Qiao, L.; Li, L.-J.; Li, S. High-κ Perovskite Membranes as Insulators for Two-Dimensional Transistors. *Nature* **2022**, *605* (7909), 262–267. https://doi.org/10.1038/s41586-022-04588-2.
(23) Puebla, S.; Pucher, T.; Rouco, V.; Sanchez-Santolino, G.; Xie, Y.; Zamora, V.; Cuellar, F. A.; Mompean, F. J.; Leon, C.; Island, J. O.; Garcia-Hernandez, M.; Santamaria, J.; Munuera, C.; Castellanos-Gomez, A. Combining Freestanding Ferroelectric Perovskite Oxides with Two-Dimensional Semiconductors for High Performance Transistors. *Nano Lett.* **2022**, *22* (18), 7457–7466. https://doi.org/10.1021/acs.nanolett.2c02395.
(24) Zhang, B.; Zhu, Y.; Zeng, Y.; Zhao, Z.; Huang, X.; Qiu, D.; Fang, Z.; Wang, J.; Xu, J.; Wang, R.; Gao, S.; Hou, Y. General Approach for Two-Dimensional Rare-Earth Oxyhalides with High Gate Dielectric Performance. *J. Am. Chem. Soc.* **2023**, *145* (20), 11074–11084. https://doi.org/10.1021/jacs.3c00401.
(25) Liu, K.; Jin, B.; Han, W.; Chen, X.; Gong, P.; Huang, L.; Zhao, Y.; Li, L.; Yang, S.; Hu, X.; Duan, J.; Liu, L.; Wang, F.; Zhuge, F.; Zhai, T. A Wafer-Scale van Der Waals Dielectric Made from an Inorganic Molecular Crystal Film. *Nat. Electron.* **2021**, *4* (12), 906–913. https://doi.org/10.1038/s41928-021-00683-w.
(26) Zou, X.; Xu, J.; Liu, L.; Wang, H.; Tang, W. M. Long-Term Stability of Multilayer MoS2 Transistors with Mica Gate Dielectric. *Nanotechnology* **2020**, *31* (18), 185202. https://doi.org/10.1088/1361-6528/ab6ab2.
(27) Mohrmann, J.; Watanabe, K.; Taniguchi, T.; Danneau, R. Persistent Hysteresis in Graphene-Mica van Der Waals Heterostructures. *Nanotechnology* **2015**, *26* (1), 015202. https://doi.org/10.1088/0957-4484/26/1/015202.
(28) Low, C. G.; Zhang, Q.; Hao, Y.; Ruoff, R. S. Graphene Field Effect Transistors with Mica as Gate Dielectric Layers. *Small* **2014**, *10* (20), 4213–4218. https://doi.org/10.1002/smll.201303929.
(29) Lee, K. H.; Shin, H.-J.; Lee, J.; Lee, I.; Kim, G.-H.; Choi, J.-Y.; Kim, S.-W. Large-Scale Synthesis of High-Quality Hexagonal Boron Nitride Nanosheets for Large-Area Graphene Electronics. *Nano Lett.* **2012**, *12* (2), 714–718. https://doi.org/10.1021/nl203635v.
(30) Dean, C. R.; Young, A. F.; Meric, I.; Lee, C.; Wang, L.; Sorgenfrei, S.; Watanabe, K.; Taniguchi, T.; Kim, P.; Shepard, K. L.; Hone, J. Boron Nitride Substrates for High-Quality

Graphene Electronics. *Nat. Nanotechnol.* **2010**, *5* (10), 722–726. https://doi.org/10.1038/nnano.2010.172.

(31) Hattori, Y.; Taniguchi, T.; Watanabe, K.; Nagashio, K. Layer-by-Layer Dielectric Breakdown of Hexagonal Boron Nitride. *ACS Nano* **2015**, *9* (1), 916–921. https://doi.org/10.1021/nn506645q.

(32) Hong, S.; Lee, C.-S.; Lee, M.-H.; Lee, Y.; Ma, K. Y.; Kim, G.; Yoon, S. I.; Ihm, K.; Kim, K.-J.; Shin, T. J.; Kim, S. W.; Jeon, E.; Jeon, H.; Kim, J.-Y.; Lee, H.-I.; Lee, Z.; Antidormi, A.; Roche, S.; Chhowalla, M.; Shin, H.-J.; Shin, H. S. Ultralow-Dielectric-Constant Amorphous Boron Nitride. *Nature* **2020**, *582* (7813), 511–514. https://doi.org/10.1038/s41586-020-2375-9.

(33) Robertson, J.; Wallace, R. M. High-K Materials and Metal Gates for CMOS Applications. *Mater. Sci. Eng. R Rep.* **2015**, *88*, 1–41. https://doi.org/10.1016/j.mser.2014.11.001.

(34) Ranjan, A.; Raghavan, N.; Holwill, M.; Watanabe, K.; Taniguchi, T.; Novoselov, K. S.; Pey, K. L.; O'Shea, S. J. Dielectric Breakdown in Single-Crystal Hexagonal Boron Nitride. *ACS Appl. Electron. Mater.* **2021**, *3* (8), 3547–3554. https://doi.org/10.1021/acsaelm.1c00469.

(35) McPherson, J. W. Increases in Lorentz Factor with Dielectric Thickness. *World J. Condens. Matter Phys.* **2016**, *6* (2), 152–168. https://doi.org/10.4236/wjcmp.2016.62018.
